# Equation of State for the Thermodynamic Properties of *Trans*-1,2-dichloroethene [R-1130(E)]

**DOI:** 10.1007/s10765-025-03535-3

**Published:** 2025-03-28

**Authors:** Marcia L. Huber, Andrei F. Kazakov, Eric W. Lemmon

**Affiliations:** Applied Chemicals and Materials Division, National Institute of Standards and Technology, Boulder, CO 80305 USA

**Keywords:** 1,2-Dichloroethene, EOS, Equation of state, Helmholtz energy, R-1130(E), Thermodynamic properties

## Abstract

We present an empirical equation of state in terms of the Helmholtz energy for *trans*-1,2-dichloroethene [R-1130(E)]. The range of validity is from the triple-point temperature, 223.31 K to 525 K with pressures up to 30 MPa. It may be used to calculate all thermodynamic properties in the fluid phase, including liquid, gas, and supercritical regions. Comparisons are given with existing literature data and estimated uncertainties are provided. In addition, checks were made for correct extrapolation behavior so that the equation behaves in a physically realistic manner when used outside of its range of validity, enabling its use in mixture models. The estimated uncertainties (at a *k* = 2 or 95 % level of confidence) are based on comparisons with critically assessed data and are 0.25 % for vapor pressure for temperatures in the range 300 K < *T* < 454 K, rising to 1.5 % as the temperature decreases from 300 K to 265 K. For density in the liquid phase the estimated uncertainty is 0.14 % for temperatures 270 K < *T* < 410 K and for pressures up to 30 MPa. For the vapor phase the estimated uncertainty in density is 3 %. The uncertainty for liquid-phase heat capacity is 1 % at atmospheric pressure over the temperature range 268 K < *T* < 309 K, and the uncertainty for the speed of sound in the liquid phase is 0.25 % for temperatures 230 K < *T* < 420 K and for pressures up to 30 MPa. The uncertainties are larger outside of these specified ranges and in the critical region.

**Supplementary Information:**

The online version contains supplementary material available at 10.1007/s10765-025-03535-3.

## Introduction

The substance *trans*-1,2-dichloroethene [R-1130(E)], formula C_2_H_2_Cl_2_, CAS 156-60-5, is a chloroolefin with low Ozone Depletion Potential (ODP) and low global warming potential (GWP), 0.00 024 and 5 respectively [1], that was added to ANSI/ASHRAE Standard 34 [2] in 2016 and previously used in applications as an industrial solvent [3]. It is currently receiving interest from the refrigeration community due to its inclusion in the azeotropic blend R-514A [74.7/25.3 mass % R-1336mzz(Z)/R-1130(E)] that is under consideration as a working fluid for centrifugal chillers, high-temperature heat pumps, and organic Rankine cycles [4]. Recently, new data have been published on the surface tension [4], thermal conductivity [5], density [4, 6, 7], speed of sound [8], and vapor pressure of R-1130(E) [4, 6, 9]. In addition, an extended corresponding states model [4] has been published for the thermodynamic properties, such as pressure-density-temperature (*pρT*) behavior. However, until now, a Helmholtz energy equation of state for this fluid has not been available. The purpose of this work is to incorporate recent measurements and develop a fundamental Helmholtz energy equation of state (EOS) for R-1130(E).

## The Helmholtz Energy Equation of State

The equation of state we present here is expressed in terms of the Helmholtz energy as a function of temperature and density. The form of the equation is

1 $$ \frac{a(T,\rho )}{{RT}} = \alpha (\tau ,\delta ) = \alpha^{{\text{o}}} (\tau ,\delta ) + \alpha^{{\text{r}}} (\tau ,\delta ), $$

where *a* is the molar Helmholtz energy, *α* is the dimensionless Helmholtz energy, *R* is the molar gas constant, *τ* = *T*_c_/*T* is the reciprocal reduced temperature, *T*_c_ is the critical temperature, *δ* = *ρ*/*ρ*_c_ is the reduced density, and *ρ*_c_ is the critical density. The Helmholtz energy in Eq. 1 is comprised of an ideal-gas contribution, *α*^o^, and a residual contribution, *α*^r^. Table 1 gives fluid-specific parameters and physical constants associated with the development of this equation. There are three measured values for the critical temperature of R-1130(E) in the literature [3, 10, 11]. McGovern [3] provides a value of 516.469 K but the manuscript states that the values are based on critical selections of available literature, sometimes supplemented by additional measurements carried out at the author’s employer and the type of apparatus and experimental details were not given. Christou [10] reported a critical temperature of 515.46 K in a study of binary mixtures, and this measurement was assessed and an uncertainty of 5 K was assigned by the NIST ThermoData Engine [12]. The most reliable measurement is that of Christou *et al.* [11], 515.5 K, performed on a sample with a purity of greater than 99.4 mol %, with an estimated uncertainty of 0.3 K [12]. The value obtained in this work, 515.69 K, is the result of the multiproperty regression procedure that employs thermodynamic constraints to ensure proper behavior in the critical region, and falls within the uncertainty band of Christou *et al.* [11]. The only reported value for *p*_c_ is from McGovern [3], mentioned earlier. No measurements of *ρ*_c_ were found, and the values for *p*_c_ and *ρ*_c_ in Table 1 were obtained by the multiproperty regression. The triple point is the result of the critical assessment of three literature sources [3, 13, 14] as recommended by the NIST ThermoData Engine [12].

**Table 1:** Constants and fluid-specific parameters for R-1130(E)

Symbol	Quantity	Value	Unit	Reference
*R*	Molar gas constant	8.314462618	J·mol^−1^·K^−1^	[15]
*M*	Molar mass	96.94328	g·mol^−1^	[16]
*T*_c_	Critical temperature	515.69	K	This work
*p*_c_	Critical pressure	5255.46	kPa	This work
*ρ*_c_	Critical density	4.54	mol·dm^−3^	This work
*T*_nbp_	Normal boiling-point temperature	320.367	K	This work
*T*_tp_	Triple-point temperature	223.31	K	[12]
*ω*	Acentric factor	0.194	–	This work

One can obtain all thermodynamic properties from taking derivatives of the Helmholtz energy in Eq. 1, as discussed in references [17] and [18]. Some particularly useful ones are

2 $$ p = \rho RT\left[ {1 + \delta \left( {\frac{{\partial \alpha^{{\text{r}}} }}{\partial \delta }} \right)_{\tau } } \right] $$

3 $$ \frac{h}{RT} = \tau \left[ {\left( {\frac{{\partial \alpha^{{\text{o}}} }}{\partial \tau }} \right)_{\delta } + \left( {\frac{{\partial \alpha^{{\text{r}}} }}{\partial \tau }} \right)_{\delta } } \right] + \delta \left( {\frac{{\partial \alpha^{{\text{r}}} }}{\partial \delta }} \right)_{\tau } + 1 $$

4 $$ \frac{{c_{v} }}{R} = - \tau^{2} \left[ {\left( {\frac{{\partial^{2} \alpha^{{\text{o}}} }}{{\partial \tau^{2} }}} \right)_{\delta } + \left( {\frac{{\partial^{2} \alpha^{{\text{r}}} }}{{\partial \tau^{2} }}} \right)_{\delta } } \right] $$

5 $$ B(T) = \mathop {\lim }\limits_{\delta \to 0} \left[ {\frac{1}{{\rho_{{\text{c}}} }}\left( {\frac{{\partial \alpha^{{\text{r}}} }}{\partial \delta }} \right)_{\tau } } \right] $$

where *p* is the pressure, *h* is the molar enthalpy, *c*_*v*_ is the isochoric heat capacity, and *B* is the second virial coefficient. Other expressions are given in references [17] and [18].

The ideal-gas Helmholtz energy *α*^o^ is given by the expression,

6 $$ \alpha^{{\text{o}}} (\tau ,\delta ) = \frac{{h_{0}^{{\text{o}}} \tau }}{{RT_{{\text{c}}} }} - \frac{{s_{0}^{{\text{o}}} }}{R} - 1 + \ln \frac{{\delta \tau_{0} }}{{\delta_{0} \tau }} - \frac{\tau }{R}\int\limits_{{\tau_{0} }}^{\tau } {\frac{{c_{p}^{{\text{o}}} }}{{\tau^{2} }}} {\text{d}}\tau + \frac{1}{R}\int\limits_{{\tau_{0} }}^{\tau } {\frac{{c_{p}^{{\text{o}}} }}{\tau }} {\text{d}}\tau , $$

where *τ*_0_ = *T*_c_/*T*_0_, *δ*_0_ = *ρ*_0_/*ρ*_c_ = *p*_0_/(*RT*_0_*ρ*_c_), *T*_0_ is an arbitrary reference state temperature, *p*_0_ is a reference pressure for the ideal gas, and *ρ*_0_ is the ideal-gas density at *T*_0_ and *p*_0_. The specific choice of reference state for this work is discussed later.

In Eq. 6, the ideal-gas heat capacity $c_{p}^{{\text{o}}}$ is required. We were unable to locate experimental data for gas-phase heat capacity, and instead used computational chemistry to obtain values for the ideal-gas heat capacity that were incorporated into the multiproperty fitting process. The ideal-gas heat capacities at constant pressure were evaluated with a conventional rigid-rotor/harmonic oscillator model with vibrational frequencies computed at the B3LYP-D3(BJ)/def2-TZVP level. The empirical frequency scaling factors [19] used in calculations were 0.960 for hydrogen stretches and 0.985 for all other modes [20]. The resulting heat capacities agree within 0.7 % for temperatures above 200 K, with the results derived from the experimental vibrational spectrum [21]. The agreement with the group-contribution scheme for chlorinated alkanes and alkenes [22] is within 0.2 % between 300 K and 1000 K. Tabulated values for $c_{p}^{{\text{o}}}$ are included in the supplemental information. We included these values in the multiproperty fitting procedure for regions where there are no experimental speed of sound data (*T* < 200 K and *T* > 500 K) and obtained the following equation,

7 $$ \frac{{c_{p}^{{\text{o}}} }}{R} = n_{0}^{{\text{o}}} + \sum\limits_{i = 1}^{3} {n_{i}^{{\text{o}}} } \left( {\frac{{m_{i}^{{\text{o}}} }}{T}} \right)^{2} \frac{{\exp (m_{i}^{{\text{o}}} /T)}}{{[\exp (m_{i}^{{\text{o}}} /T) - 1]^{2} }}, $$

where the coefficients $n_{i}^{{\text{o}}}$ and the exponents $m_{i}^{{\text{o}}}$ are presented in Table 2.

**Table 2:** Parameters of Eqs. 7 and 8 for R-1130(E)

*i*	*n*_*i*_^o^	*m*_*i*_^o^
0	4	–
1	2.697	367 K
2	6.264	1246 K
3	3.041	4030 K
4	− 14.91 185 845 478 433	–
5	8.646 546 650 620 836	–

The ideal-gas Helmholtz energy derived from Eqs. 6 and 7 may be written as [23]

8 $$ \alpha^{{\text{o}}} (\tau ,\delta ) = \ln \delta + n_{4}^{{\text{o}}} + n_{5}^{{\text{o}}} \tau + (n_{{0}}^{{\text{o}}} - 1)\ln \tau + \sum\limits_{i = 1}^{3} {n_{{\text{i}}}^{{\text{o}}} } \ln \left[ {1 - \exp \left( { - \frac{{m_{{\text{i}}}^{{\text{o}}} \tau }}{{T_{{\text{c}}} }}} \right)} \right] $$

with $n_{i}^{{\text{o}}}$ and $m_{i}^{{\text{o}}}$ given in Table 2. These values were determined according to the reference-state convention of the International Institute of Refrigeration (IIR), that the specific enthalpy and entropy of the saturated liquid state at 0 °C are 200 kJ·kg^−1^ and 1 kJ·kg^−1^·K^−1^, respectively. Users interested in defining other reference states may find the enthalpies of formation in Manion’s work [24] useful. The large number of digits in $n_{4}^{{\text{o}}}$ and $n_{5}^{{\text{o}}}$ are necessary to accurately reproduce the reference state values.

The residual Helmholtz energy was fit to the following empirical equation [23],

9 $$ \alpha^{{\text{r}}} {(}\tau {,}\delta {) = }\sum\limits_{i = 1}^{{K_{1} }} {n_{i} } \tau^{{t_{i} }} \delta^{{{\text{d}}_{i} }} + \sum\limits_{{i = K_{1} + 1}}^{{K_{2} }} {n_{i} \tau^{{t_{i} }} \delta^{{d_{i} }} \exp ( - g_{i} \delta^{{e_{i} }} ) + \sum\limits_{{i = K_{2} + 1}}^{{K_{3} }} {n_{i} \tau^{{t_{i} }} \delta^{{d_{i} }} \exp [ - \eta_{i} (\delta - \varepsilon_{i} )^{2} - \beta_{i} (\tau - \gamma_{i} )^{2} ]} ,} $$

where the number of terms *K*_1_, *K*_2_, and *K*_3_ and the values of the coefficients *n*_*i*_, exponents *t*_*i*_ and *d*_*i*_, parameters *g*_*i*_ and *e*_*i*_, and Gaussian parameters *η*_*i*_, *ε*_*i*_, *β*_*i*_, and *γ*_*i*_ are determined by fitting experimental data in a multiproperty fitting procedure that incorporates a variety of fluid properties such as the density, vapor pressure, critical parameters, heat capacity, and speeds of sound. For refrigerants, *g*_*i*_ has been typically set to 1 [25–28], but, following the recent work of Akasaka and Lemmon [23], we have included it in the fit. The first set of terms are polynomials, the second set contain exponentials, and the third set are often called the Gaussian bell-shaped terms.

The regression procedure utilized an in-house nonlinear fitting algorithm originally developed by Lemmon and Jacobsen [17]. Over the years it has been updated and improved following collaborations with many researchers [25, 28–36] and is used extensively for fitting Helmholtz-energy equations of state. We followed the procedure described most recently by Akasaka and Lemmon [23], that includes the specific details. The resulting parameters are given in Table 3. The large number of digits for *n*_*5*_ and *n*_*6*_ are required to satisfy the critical conditions. Of note is that in contrast to the recent EOS for R-1243zf of Akasaka and Lemmon [23], all of our *β*_*i*_ values are zero. However, we leave them in our table for easier comparisons with previous EOS models.

**Table 3:** Parameters of Eq. 9 for the residual part of the Helmholtz energy for R-1130(E)

*i*	*n*_*i*_	*t*_*i*_	*d*_*i*_	*e*_*i*_	*g*_*i*_	*η*_*i*_	*β*_*i*_	*γ*_*i*_	*ε*_*i*_
1	0.0256	1	4						
2	0.9	0.188	1						
3	0.054	0.2496	2						
4	0.153	0.458	3						
5	– 0.49 787 364 754	0.77	1						
6	– 0.99 000 024 872	1.0385	2						
7	– 0.318	0.9	1	1	2.43				
8	– 0.973	1.21	1	2	1.236 548				
9	– 0.098	4.7	1	3	0.722				
10	– 0.034	6.61 575	2	3	1.483				
11	– 0.0971	2.257	3	3	0.2255				
12	– 0.26	3.2664	1			5.10 245	0	1	– 0.31 662
13	– 0.135	3.59	2			4.572	0	1	0.065
14	– 0.21	1.2	1			4.63	0	1	– 0.28 568
15	– 0.197	1.42	1			1.785	0	1	0.99

Test values for checking computer implementation of the full EOS are provided in Table 4, where *w* is the speed of sound, *c*_*p*_ and *c*_*v*_ are the heat capacity at constant pressure and volume, respectively, and *h* is the enthalpy. In the supporting information we provide files that can be used to reproduce these numbers.

**Table 4:** Test values for verification of computer implementation of the EOS for R-1130(E)

*T* (K)	*ρ* (mol·dm^−3^)	*p* (MPa)	*w* (m·s^−1^)	*c*_*p*_ (J·mol^−1^·K^−1^)	*c*_*v*_ (J·mol^−1^·K^−1^)	*h* (J·mol^−1^)
320	0.0	0.00 000	176.452	70.151	61.836	52 896.5
320	0.02	0.05 229	174.174	71.732	62.647	52 732.4
320	12.5	3.39 671	946.434	115.586	76.665	24 883.3
400	0.02	0.06 584	194.273	79.550	70.843	58 759.7
400	11.0	5.40 729	672.429	124.461	81.788	34 519.1
520	9.0	19.12 429	454.513	136.581	88.434	50 110.9

In order to determine the saturation conditions of the EOS, the Maxwell criterion [37] is applied. This is an iterative process and can be computationally expensive. To speed up these calculations, ancillary equations for the vapor pressure and the saturated liquid and saturated vapor densities were developed to provide accurate starting guesses for iterative procedures. The equation for vapor pressure *p*_s_ is

10 $$ \ln \left( {\frac{{p_{{\text{s}}} }}{{p_{{\text{c}}} }}} \right) = \left( {\frac{{T_{{\text{c}}} }}{T}} \right)\sum\limits_{i = 1}^{5} {n_{i} } \left( {1 - \frac{T}{{T_{{\text{c}}} }}} \right)^{{t_{i} }} , $$

and the equations for saturated liquid density *ρ*′ and saturated vapor density *ρ*″ are

11 $$ \frac{{\rho^{\prime}}}{{\rho_{{\text{c}}} }} = 1 + \sum\limits_{i = 1}^{6} {n_{i} \left( {1 - \frac{T}{{T_{{\text{c}}} }}} \right)}^{{t_{i} }} $$

and

12 $$ \frac{{\rho^{\prime\prime}}}{{\rho_{{\text{c}}} }} = \sum\limits_{i = 1}^{7} {n_{i} \left( {1 - \frac{T}{{T_{{\text{c}}} }}} \right)}^{{t_{i} }} . $$

**Fig. 1:**
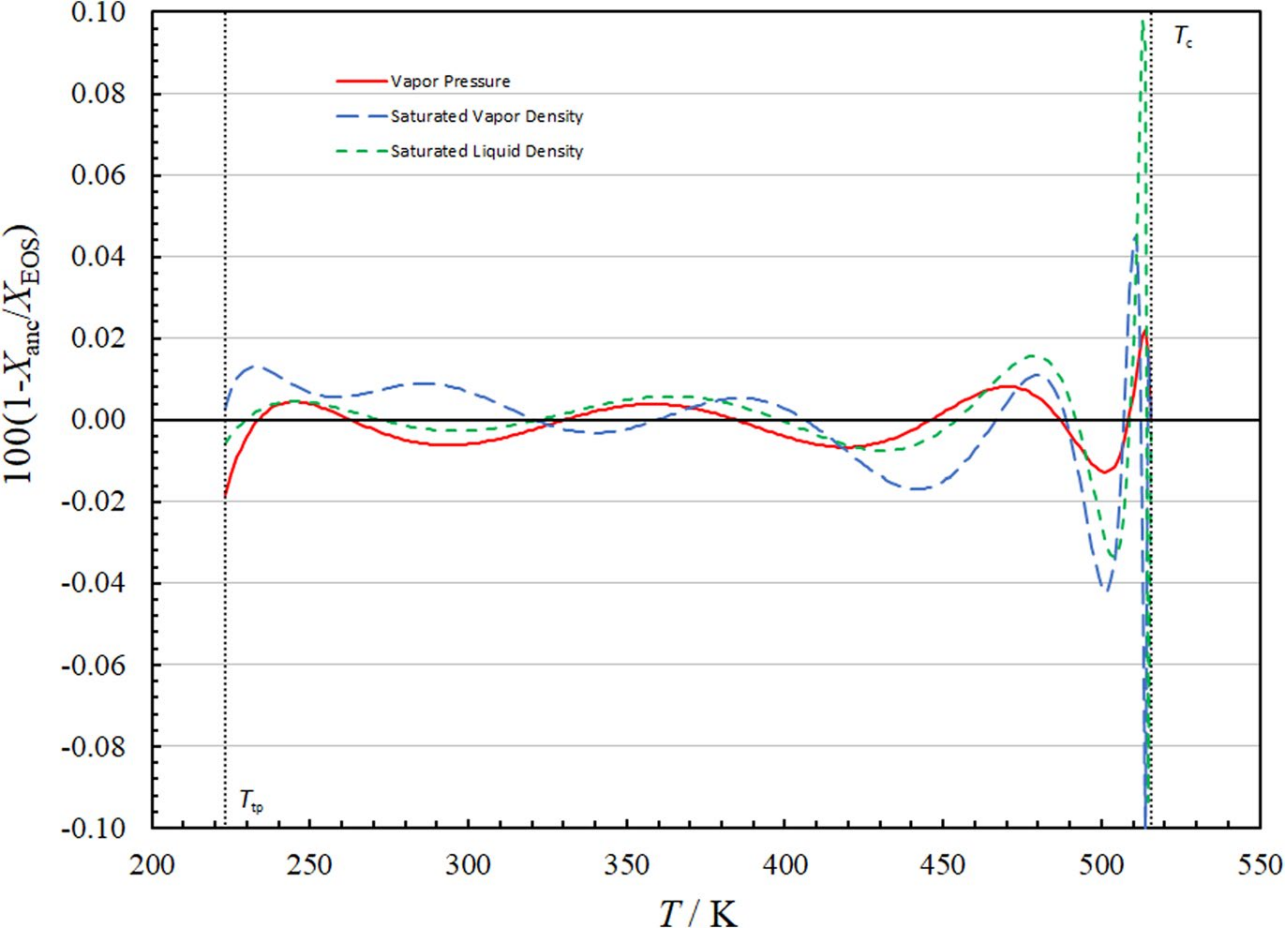
Relative deviations in vapor pressures, saturated liquid densities, and saturated vapor densities calculated with the ancillary equations from the Maxwell solution of the EOS

We fitted the data obtained by rigorously computing the Maxwell solutions for the saturation properties of the EOS. The values of the parameters for the ancillary equations are given in Table 5. Figure 1 shows the relative deviations in vapor pressures and saturated liquid and saturated vapor densities calculated with the ancillary equations from the Maxwell solution of the EOS. These correlations may be used to estimate starting values, but for the actual calculation of vapor–liquid-equilibrium, the full EOS subject to the Maxwell criterion should be used.

**Table 5:** Parameters of Eqs. 10–12 for the ancillary equations for R-1130(E)

_*i*_	Vapor pressure, Eq. 10		Saturated liquid density, Eq. 11		Saturated vapor density, Eq. 12	
	*n*_*i*_	*t*_*i*_	*n*_*i*_	*t*_*i*_	*n*_*i*_	*t*_*i*_
1	–7.2526	1	4.577	0.47	–0.063 124	0.05
2	3.8005	1.5	–6.1201	0.83	–10.541	0.64
3	–7.7286	2.12	7.6048	1.23	23.782	1.13
4	10.953	2.84	–4.7251	1.69	–56.858	1.74
5	–9.7064	3.58	1.6728	2.27	82.754	2.57
6			1.8053	9.8	–76.847	3.25
7					–70.617	8.66

## Comparisons with Experimental Data

**Fig. 2:**
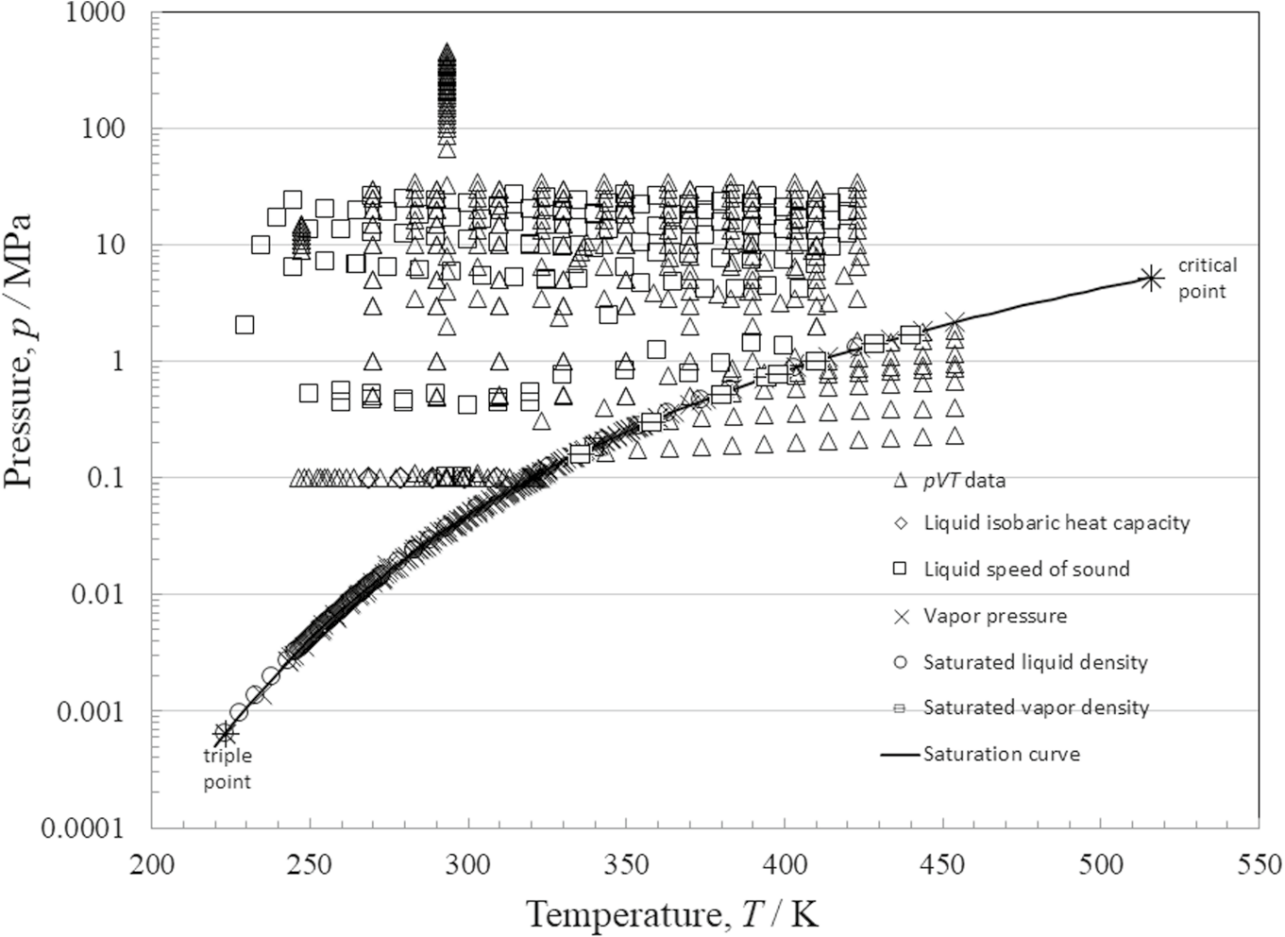
*p*,*T* diagram of available experimental data for R-1130(E)

Table 6 lists the experimental thermodynamic data for R-1130(E). We made extensive use of the NIST ThermoData Engine [12] to identify data sources. Table 6 summarizes, to the best of our knowledge, all the available experimental thermodynamic property measurements for R-1130(E) reported in the literature. It also provides the experimental method, the sample purity, the uncertainty as reported by the original authors, the number of measurements, and the ranges of temperatures, pressures, and densities. The uncertainty in Table 6 is at the 95 % confidence (*k* = 2) level. Unfortunately, many authors often failed to report *k*; we assumed *k* = 2 for those cases. The values are as reported by the authors, and at times may be overly optimistic. All discussions of uncertainty in this work will be at the 95 % level of confidence. Figure 2 illustrates the distribution of the different types of property data in *p*–*T* space. In Table 6 we also summarize the results of comparisons with the experimental data. We use the following expressions for the percent deviation (PCT), average absolute relative deviation (AARD), BIAS, and the standard deviation (STDEV),

13 $$ {\text{PCT}}_{i} = \frac{{100\;\Delta X_{i} }}{{X_{i} }} = \frac{{100(X_{\exp ,i} - X_{{\text{calc,i}}} )}}{{X_{\exp ,i} }}, $$

14 $$ {\text{AARD = }}\left( {\sum\limits_{i = 1}^{n} {\left| {{\text{PCT}}_{i} } \right|} } \right)/n, $$

15 $$ {\text{BIAS = }}\left( {\sum\limits_{i = 1}^{n} {{\text{PCT}}_{i} } } \right)/n,\quad {\text{and}} $$

16 $$ {\text{STDEV = }}\sqrt {({\text{PCT}}_{i} - {\text{BIAS}})^{2} /n} , $$

where *n* is the number of data points and *X* is an arbitrary thermodynamic property.

**Table 6:** Summary and comparisons with experimental data for R-1130(E)

1st Author (year)	References	No. data	Method	Purity	Unc. (%)	*T* (K)	*p* (MPa)	AARD (%)	BIAS (%)	STDEV (%)	Max (%)
Liquid-phase isobaric heat capacity											
Straka (2012)	[38]	20	FC	0.997^a^	1	268–309	0.1	0.43	− 0.33	0.46	− 1.3
Liquid-phase speed of sound											
Dimitriu (1982)	[39]	1	Opt	na	na	293	0.1	0.02	− 0.02	–	− 0.02
Lienert (1975)	[40]	1	UI	na	na	298	0.1	0.2	− 0.2	–	− 0.2
Rowane (2024)	[8]	145	DPE	0.991^a^	0.03–0.06	230–420	0.2–26.7	0.1	0.00	0.12	0.25
Vapor pressure											
Akasaka (2025)	[41]	16	IsoPVT	0.9960^b^	na	300–375	0.048–0.476	0.12	0.07	0.11	0.24
Amaya (1961)	[42]	1	na	na	na	320	0.1	0.21	− 0.21	–	− 0.21
Andrews (1951)	[43]	1	na	na	na	321	0.1	3.3	− 3.3	–	− 3.3
Awbery (1936)	[44]	1	na	> 0.90^b^	na	323	0.1	7.9	− 7.9	–	− 7.9
Flom (1951)	[45]	11	ES	na	0.12–0.15	287–325	0.03–0.11	5.3	− 5.3	0.33	− 5.8
Giles (2006)	[46]	2	CCS	0.9985^b^	0.2–0.9	303–353	0.05–0.27	1.6	− 1.6	0.58	− 2.0
Herz (1913)	[47]	9	na	na	na	296–322	0.04–0.1	5.0	− 5.0	0.49	− 5.9
Hiaki (2002)	[48]	1	ES	0.999^a^	0.1	320	0.1	1.4	− 1.4	–	− 1.4
Hsia (1931)	[49]	11	Dis	na	na	244–296	0.003–0.04	5.2	− 5.2	2.1	− 6.9
Hsia (1931)	[50]	19	Dis	na	2–3	243–333	0.003–0.15	4.4	− 4.3	1.7	− 6.3
Ketelaar (1947)	[51]	44	GST	na	0.04–5	235–358	0.001–0.30	1.8	− 0.89	2.9	13.0
Kovac (1985)	[52]	2	ES	na	0.3	320	0.1	0.25	− 0.25	0.02	− 0.26
Lombardo (2023)	[6]	36	ES	0.997^b^	0.05–0.25	283–353	0.026–0.276	1.7	1.7	1.1	3.7
Machat (1985)	[53]	12	EB	0.999^a^	0.04–0.28	272–320	0.014–0.098	1.8	− 1.8	0.2	− 2.1
Mato (1991)	[54]	1	ES	0.9985	0.12	313	0.08	2.4	− 2.4	–	− 2.4
Mumford (1950)	[55]	1	na	na	na	321	0.1	4.0	− 4.0	–	− 4.0
Newitt (1951)	[56]	1	na	na	na	321	0.1	3.5	− 3.5	–	− 3.5
Putze (1995)	[57]	1	CCS	0.99^a^	na	298	0.044	2.1	− 2.1	–	− 2.1
Rowane (2024)	[9]	32	CCS	0.991^a^	0.12–2.23	265–360	0.01–0.33	0.28	− 0.08	0.37	− 1.2
Sagnes (1971)	[58]	1	EB	0.998	na	321	0.1	0.58	− 0.58	–	− 0.58
Tanaka (2022)	[4]	14	IsoPVT	0.997^b^	0.3	324–454	0.11–2.2	0.74	− 0.58	0.88	− 2.3
Volman (1948)	[59]	1	na	na	na	322	0.1	3.48	− 3.48	–	− 3.48
Saturated liquid density											
Kawanishi (1982)	[60]	56	GD,dig	na	na	246–273	sat	1.23	− 1.23	0.48	− 2.05
Ketelaar (1947)	[61]	15	Dil,EQ	na	0.05	223–293	sat	0.11	− 0.09	0.10	− 0.25
Lombardo (2023)	[6]	8	VibT	0.997^b^	0.05	283–423	sat	0.37	− 0.35	0.62	− 1.7
Tanaka (2022)	[4]	6	IsoPVT	0.997^b^	0.05–0.11	326–408	sat	0.58	− 0.58	0.26	− 0.93
Saturated vapor density											
Tanaka (2022)	[4]	8	IsoPVT	0.997^b^	0.4–3.9	335–440	sat	1.20	0.15	1.4	2.1
Second virial coefficient											
Fogg (1955)	[62]	5	BL,EQ	na	na	293–373		6.37	6.37	1.20	7.73
Single-phase density											
Awbery (1936)	[44]	1	na	> 0.90^b^	na	289	0.1	0.27	0.27	–	0.27
Belikov (2025)	[63]	14	Xrad	0.98^b^	0.07–0.19	247	9–15	0.16	− 0.16	0.03	− 0.20
Comelli (1995)	[64]	1	VibT	0.98^a^	0.001	298	0.1	0.44	0.44	–	0.44
Comelli (1991)	[65]	6	Pyc	0.98^a^	0.01	290–306	0.1	0.42	0.42	0.03	0.45
Curran (1950)	[66]	3	Pyc	na	na	298	0.1	0.12	0.12	0.01	0.12
Fortin (2024)	[7]	136	VibT	0.991^a^	0.04–0.05	270–410	0.5–30	0.11	0.11	0.07	0.21
Francesconi (1994)	[67]	1	VibT	0.98^a^	na	298	0.1	0.42	0.42	–	0.42
Francesconi (1995)	[68]	1	VibT	0.98^a^	na	298	0.1	0.42	0.42	–	0.42
Hahn (1996)	[69]	2	VibT	0.994^a^	0.0008	293–313	0.1	0.16	0.16	0.11	0.24
Hahn (1996)	[70]	6	VibT	0.994^a^	0.0008	293	0.1–10	0.22	0.22	0.01	0.23
Herz (1913)	[47]	4	na	na	na	288–318	0.1	0.34	0.34	0.12	0.48
Kawanishi (1982)	[71]	35	GD,dig	na	na	293	32–460	3.34	− 3.34	1.23	− 5.74
Kovac (1985)	[52]	1	na	na	na	293	0.1	0.23	0.23	–	0.23
Lombardo (2023)	[6]	80	VibT	0.997^b^	0.05	283–423	0.1–35	0.33	− 0.32	0.54	− 1.9
Mato (1991)	[54]	1	ES	0.9985^a^	0.0008	298	0.1	1.4	− 1.4	–	− 1.4
Mumford (1950)	[55]	2	WCSGB	na	na	293–298	0.1	0.38	0.38	0.03	0.41
Tanaka (2022)^c^	[4]	73	IsoPVT	0.997^b^	0.06–4.0	329–454	0.17–11	1.2	− 1.0	1.4	− 5.2
Volman (1948)	[59]	4	WB,dig	na	na	298–321	0.1	0.36	0.36	0.10	0.52
Zegrodnik (1989)	[72]	17	Pyc,dig	na	na	246–293	0.1	0.21	− 0.21	0.05	− 0.30

*BL* Boyles Law apparatus; *CCS* closed cell static method; *Dig* digitized from a plot; *Dil* dilatometer; *Dis* distillation; *DPE* dual path pulse echo; *EB* ebulliometer; *EQ* presented as an equation; *ES* equilibrium still; *FC* flow calorimetry; *GD* glass dilatometer; *GST* glass spring tensiometer; *IsoPVT* isochoric PVT apparatus; *Opt.* optical method; *Pyc* pycnometer; *UI* ultrasonic interferometer; *VibT* vibrating tube; *WB* Westphal balance; *WCSGB* water-calibrated specific gravity bottle; *Xrad* X-ray radiography; *na* not available

^a^Purity on mole fraction basis

^b^Purity on mass fraction basis (if basis is not given, there is no superscript)

^c^Includes both liquid and vapor points

### Comparisons with Experimental Data: Liquid-Phase Isobaric Heat Capacity

**Fig. 3:**
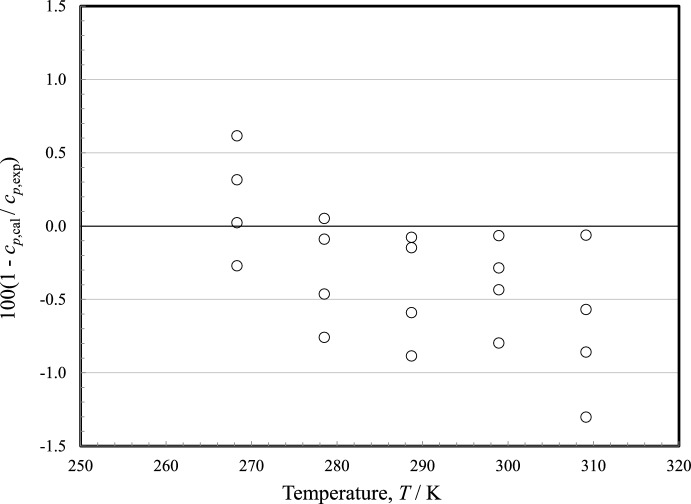
Relative deviations of the experimental data from values calculated by the EOS for liquid-phase heat capacity. Straka *et al.* [38] (○)

We found only one source of heat-capacity data, the liquid-phase measurements of Straka *et al.* [38] performed at atmospheric pressure and covering the temperature range 268 K to 309 K. One additional source of possible data in the literature was located, McGovern [3], who gave values for numerous properties (including liquid heat capacity, vapor pressure, heat of vaporization, liquid density, critical point) but no experimental details are given and it is not clear if the values are experimental, recommendations, or taken from other sources. Consequently, the data from McGovern are not included in our summary. Deviations of the liquid-phase heat capacity data are shown in Fig. 3. Straka *et al.* [38] performed their measurements with a Tian-Calvet calorimeter and gave a combined expanded uncertainty of their measurements as 1 %. The EOS represents the liquid-phase heat capacity to within this level as shown in Fig. 3.

### Comparisons with Experimental Data: Liquid-Phase Speed of Sound

**Fig. 4:**
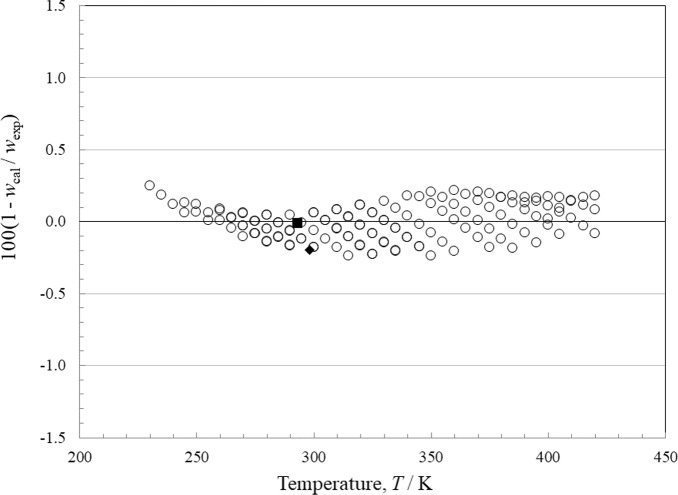
Relative deviations of the experimental data from values calculated by the EOS for liquid-phase speed of sound as a function of temperature. Dimitriu and Dima [39] (■), Lienert [40] (♦), Rowane [8] (○)

**Fig. 5:**
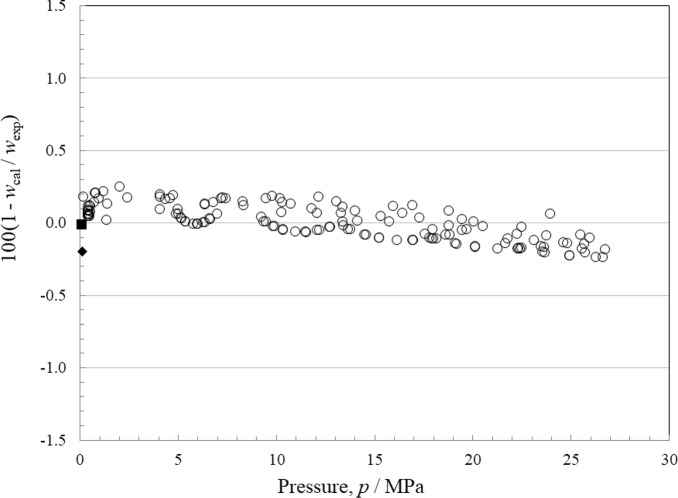
Relative deviations of the experimental data from values calculated by the EOS for liquid-phase speed of sound as a function of pressure. Dimitriu and Dima [39] (■), Lienert [40] (♦), Rowane [8] (○)

There are 3 sources of data for speed of sound in the liquid phase, and there are none for the vapor. In the liquid, Dimitriu and Dima [39] and Lienert [40] give only a single point at atmospheric pressure. Rowane [8] provides a more comprehensive set that covers the temperature range 230 K to 420 K at pressures from 0.2 MPa to 26.7 MPa. Relative deviations in the equation of state compared to the liquid-phase heat capacity data as functions of temperature and of pressure are shown in Figs. 4 and 5, respectively. At a level of *k* = 2, the data are represented to within 0.25 %, with an AARD of 0.1 %. This is significantly larger than the uncertainty stated by Rowane [8]; we feel some of this may be due to the purity of the sample, which was only 99.1 % and said to possibly contain a hydrocarbon contaminant. The data of Dimitriu and Dima [39] and also that of Lienert [40] are in very good agreement with Rowane *et al.* [8].

### Comparisons with Experimental Data: Vapor Pressure

**Fig. 6:**
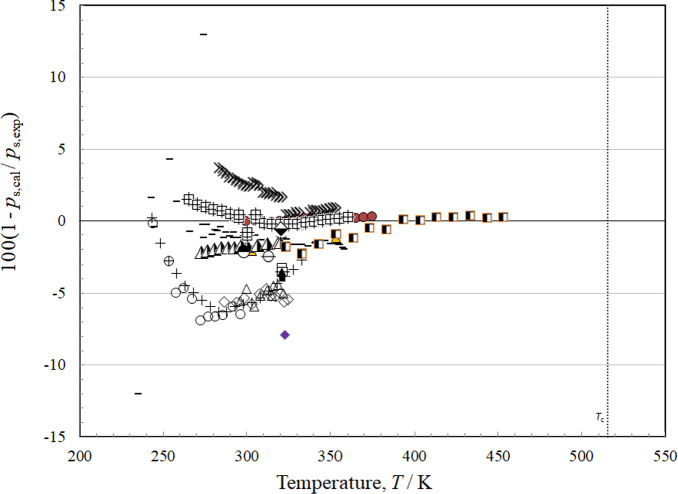
Relative deviations of the experimental data from values calculated by the EOS for vapor pressure. Akasaka *et al.* [41] (), Amaya [42] (), Andrews and Keefer [43] (□), Awbery and Griffiths [44] (♦), Flom [45] (◊), Giles and Wilson [46] (), Herz and Rathmann [47] (∆), Hiaki *et al.* [48] (×),Hsia [49] (○), Hsia [50] (+), Ketelaar *et al.* [51] (—), Kovac *et al.* [52] (), Lombardo *et al.* [6] (), Machat and Boublik [53] (), Mato and Berro [54] (), Mumford and Phillips [55] (■), Newitt and Weale [56] (), Putze *et al.* [57] (), Rowane and Outcalt [9] (), Sagnes and Sanchez [58] (), Tanaka *et al.* [4] (), Volman and Andrews [59] (▲)

**Fig. 7:**
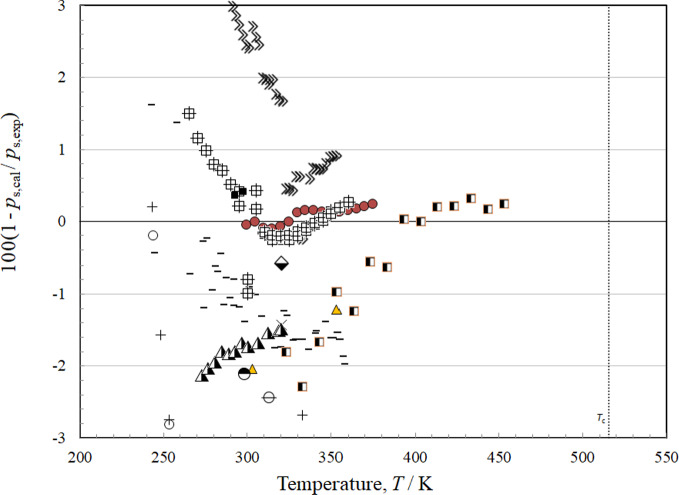
Relative deviations of the experimental data from values calculated by the EOS for vapor pressure, limited to 3 % deviations. Akasaka *et al.* [41] (), Amaya [42] (), Giles and Wilson [46] (), Hiaki *et al.* [48] (×), Hsia [49] (○), Hsia [50] (+), Ketelaar *et al.* [51] (—), Kovac *et al.* [52] (), Lombardo *et al.* [6] (), Machat and Boublik [53] (), Mato and Berro [54] (), Mumford and Phillips [55] (■), Putze *et al.* [57] (), Rowane and Outcalt [9] (), Sagnes and Sanchez [58] (), Tanaka *et al.* [4] ()

There are 22 sources of data for vapor pressure as summarized in Table 6, with a scatter of mostly within about 5 % as indicated in Fig. 6. The most comprehensive sets are those of Hsia [49, 50], Ketelaar *et al.* [61], Akasaka *et al.* [41], Flom *et al.* [45], Lombardo *et al.* [6], Machat and Boublik [53], Rowane and Outcalt [9], and Tanaka *et al.* [4]. In Hsia [49], the identities of *cis* and *trans* isomers are apparently switched and we corrected for that. In Hsia [50], the isomers are correctly identified. The isomers in Mumford and Phillips [55] also seem to have been switched, and also were corrected. Flom *et al.* [45] is in agreement with Hsia [49, 50], but these data appear to be offset from the others. Ketelaar *et al.* [51] displays large scatter at temperatures below 275 K, but otherwise agrees with the other data. The only data at high temperatures (*T* > 375 K) are from Tanaka *et al.* [4], although the highest data are still considerably below the critical temperature. Figure 7 shows the vapor pressure deviations but on a more limited scale. There is excellent agreement between the data of Akasaka *et al.* [41] and the data of Rowane and Outcalt [9] for the temperature range 300 K < *T* < 375 K, and the uncertainty in this region is 0.25 %, the uncertainty is also 0.25 % up to the limit of experimental data, 454 K. As the temperature decreases, the uncertainty increases to about 1.5 % at 265 K.

### Comparisons with Experimental Data: Saturation Density

**Fig. 8:**
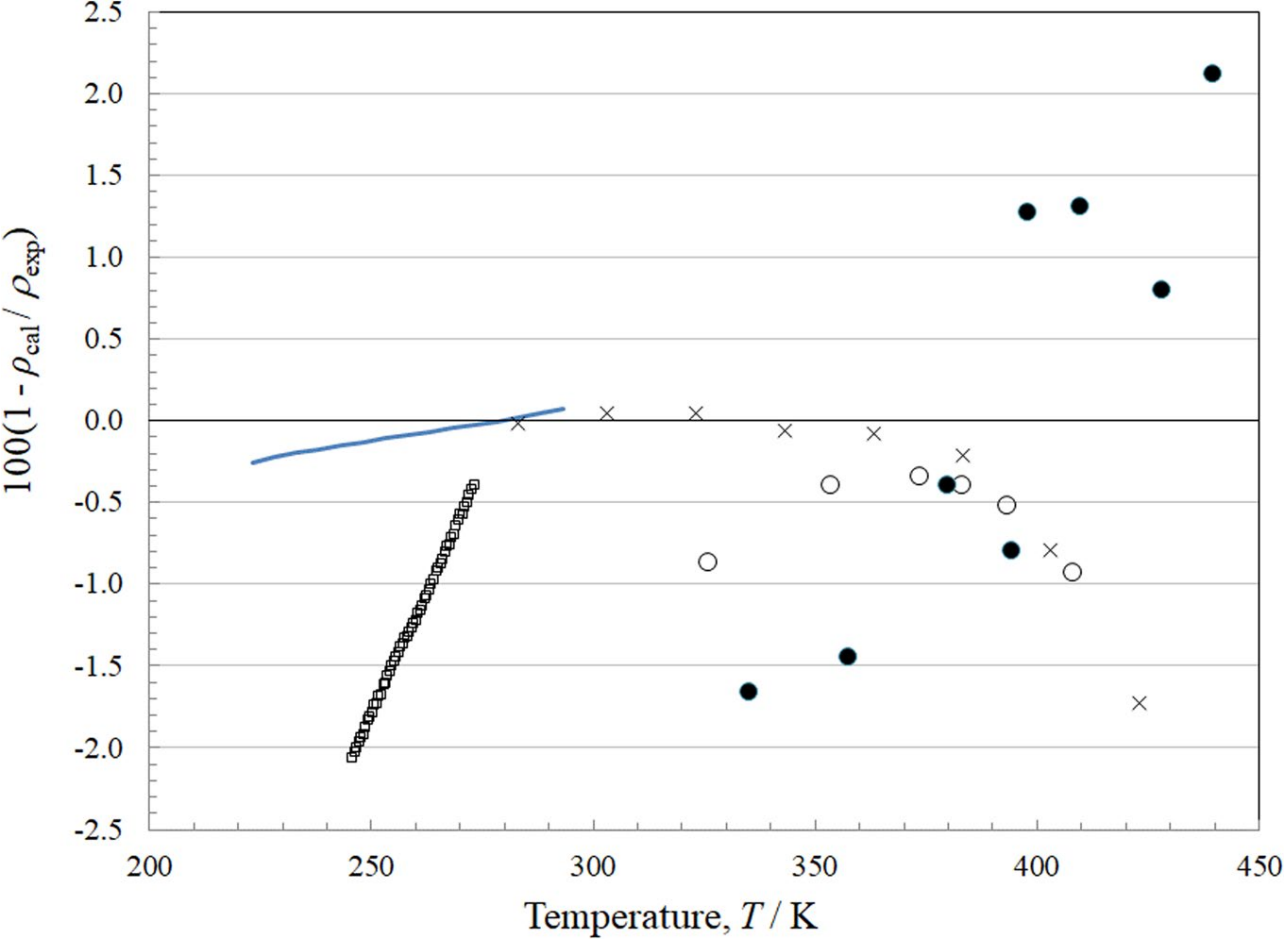
Relative deviations of the experimental data from values calculated by the EOS for density at saturation conditions. Kawanishi *et al.* [60] (▫), Ketalaar *et al.* [51] (solid line) saturated liquid, Lombardo *et al.* [6] (×) saturated liquid, Tanaka *et al.* [4] saturated liquid (○), Tanaka *et al.* [4] saturated vapor (●)

There are four sources of experimental data for the density of the saturated liquid, and one for the density of saturated vapor. The results of Ketelaar *et al.* [61] are presented only as an equation, but in the region where they overlap Lombardo *et al.* [6] the data agree well, as shown in Fig. 8. At temperatures above about 383 K the deviations increase. The data of Kawanishi *et al.* [60] were obtained by digitizing a plot, and show an odd temperature dependence. The saturated vapor densities are represented to within 2 %, but display a much larger scatter than those in the saturated liquid, and come from only one source, Tanaka *et al.* [4].

### Comparisons with Experimental Data: Density

**Fig. 9:**
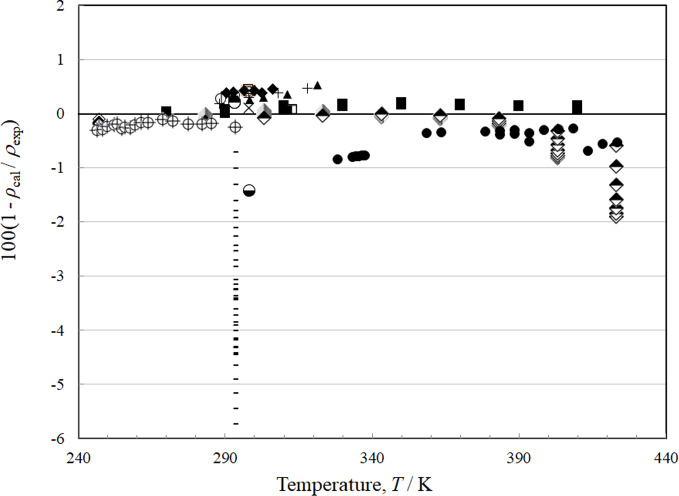
Relative deviations of the experimental data from values calculated by the EOS for density as a function of temperature for the liquid phase. Awberry and Griffiths [44] (), Belikov *et al.* [63] (◊), Comelli and Francisconi [65] (□), Comelli and Francesconi [64] (♦), Curran and Estok [66] (×), Fortin and Outcalt [7] (■), Francesconi and Comelli [67] (∆), Francesconi and Comelli [68] (), Hahn and Svejda [69] (), Hahn *et al.* [70] (), Herz and Rathmann [47] (+), Kawanishi *et al.* [71] (-), Kovac *et al.* [52] (—), Lombardo *et al.* [6] (), Mato and Berro [54] (), Mumford and Phillips [55] (○), Tanaka *et al.* [4] (●), Volman and Andrews [59] (▲), Zegrodnik *et al.* [72] ()

**Fig. 10:**
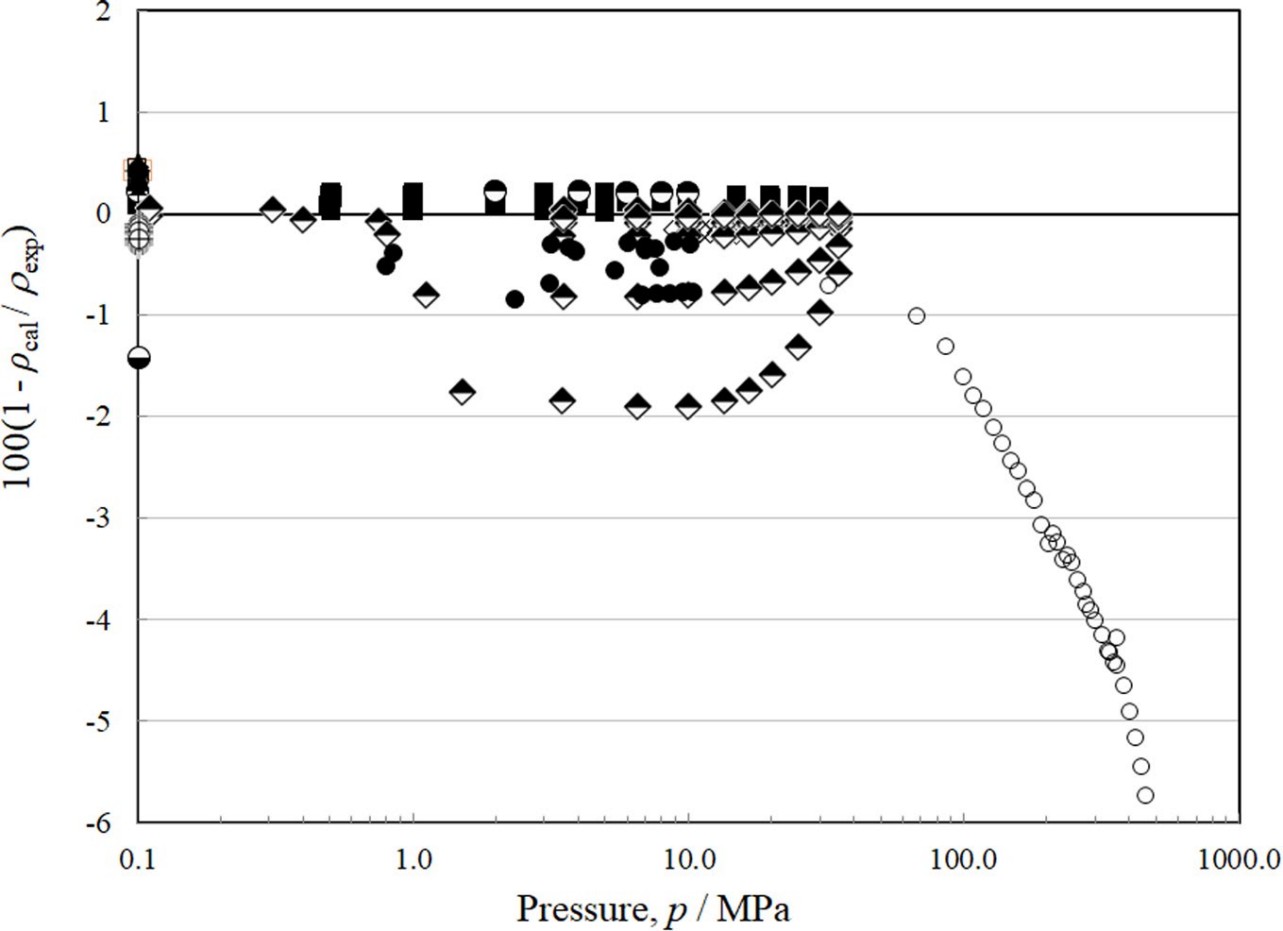
Relative deviations of the experimental data from values calculated by the EOS for density as a function of pressure for the liquid phase. Awberry and Griffiths [44] (), Belikov *et al.* [63] (◊), Comelli and Francisconi [65] (□), Comelli and Francesconi [64] (♦), Curran and Estok [66] (×), Fortin and Outcalt [7] (■), Francesconi and Comelli [67] (∆), Francesconi and Comelli [68] (), Hahn and Svejda [69] (), Hahn *et al.* [70] (), Herz and Rathmann [47] (+), Kawanishi *et al.* [71] (-), Kovac *et al.* [52] (—), Lombardo *et al.* [6] (), Mato and Berro [54] (), Mumford and Phillips [55] (○), Tanaka *et al.* [4] (●), Volman and Andrews [59] (▲), Zegrodnik *et al.* [72] ()

**Fig. 11:**
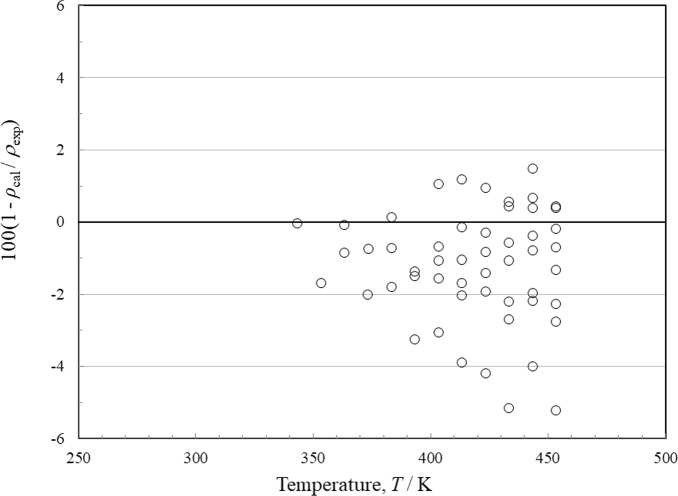
Relative deviations of the experimental data from values calculated by the EOS for density as a function of temperature for the vapor phase. Tanaka *et al.* [4] (○)

**Fig. 12:**
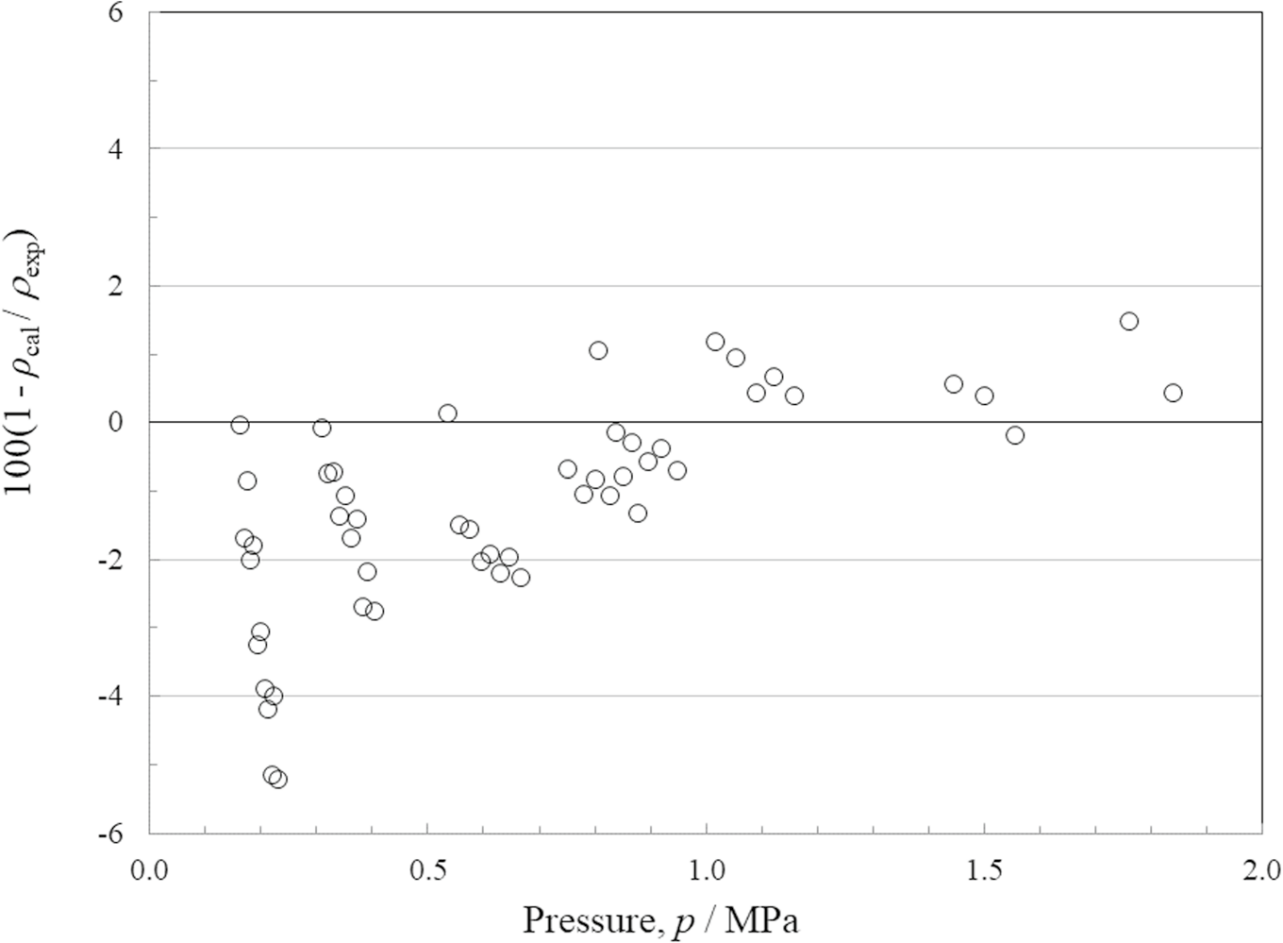
Relative deviations of the experimental data from values calculated by the EOS for density as a function of pressure for the vapor phase. Tanaka *et al.* [4] (○)

There are 19 sources of experimental single-phase density data, although only Tanaka *et al.* [4] have vapor densities. The deviations for the liquid phase as a function of temperature are shown in Fig. 9 and with respect to pressure in Fig. 10. Figures 11 and 12 show the deviations for the vapor phase. The most comprehensive and reliable sets are the most recent data obtained in 2022–2024 by Tanaka *et al.* [4], Lombardo *et al.* [6], and Fortin and Outcalt [7]. Tanaka *et al.* [4] data display an offset from Lombardo *et al.* [6] and Fortin and Outcalt [7] and Tanaka *et al.* [4] have more scatter. The data of Hahn *et al.* [70] are taken at a single temperature of 293 K but cover a range of pressures from 0.1 MPa to 10 MPa and are in excellent agreement with Fortin and Outcalt [7]. At temperatures above 383 K, the Lombardo *et al.* [6] data show increasing deviations. Fortin and Outcalt [7] suggest that possible differences in calibration could contribute to some, but not all, of the discrepancies observed. Sample purity may also be a contributing factor in some of the discrepancies seen. Based on comparisons primarily with Fortin and Outcalt [7], the estimated uncertainty in liquid density is 0.14 % at pressures up to 30 MPa over the temperature range 270 K to 410 K. As shown in Figs. 10 and 11, the estimated uncertainty in the vapor phase is much larger; the AARD for the vapor points is 1.5 % and the estimated uncertainty is 3.1 %.

The data of Kawanishi *et al.* [71] obtained with a glass dilatometer at a single temperature extend to extreme pressures, up to 460 MPa, and show deviations of almost 6 % at 460 MPa. This data set was presented only graphically in the original manuscript, and no information on sample purity or experimental uncertainty was presented; these data were not used in the regression process but are included for comparison purposes. Our primary interest in this work is to provide an EOS that can be used for practical applications in the refrigeration industry, especially as a component in the refrigerant mixture R-514A used in low-pressure centrifugal chillers, so very high pressures are not our focus. It is of interest however to note that Kawanishi *et al.* [60, 71] reported that they observed a novel liquid–liquid (LL) transition at about 200 MPa and 20 °C based on changes in the slope of the specific volume vs pressure curve, and additional liquid–liquid transition around − 16 °C at atmospheric pressure based on spin–lattice relaxation time measurements. Merkel *et al.* [73] also reported a LL transition based on IR vibrational spectroscopy studies, but recently Belikov *et al.* [63] studied the density of R-1130(E) over a temperature range from − 26 °C to 6 °C at pressures up to 15 MPa and did not observe any indication of a liquid–liquid transition. Turton *et al.* [74] also did not find evidence for this transition. Our EOS is not able to represent this behavior and based on our analysis of thermophysical property data used to develop the EOS, we did not observe any unusual behavior. However further studies may be necessary to resolve this issue.

### Comparisons with Experimental Data: Second Virial Coefficient

**Fig. 13:**
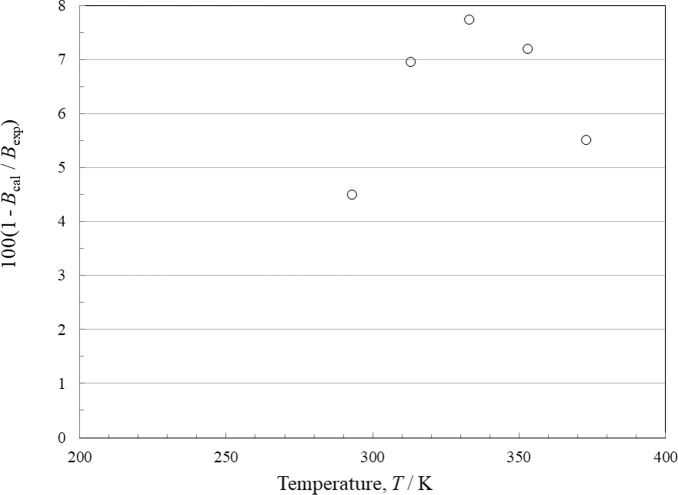
Relative deviations of the experimental data from values calculated by the EOS for the second virial coefficient. Fogg and Lambert [62] (○)

Data for the second virial coefficient are quite limited. Fogg and Lambert in 1955 [62] presented a correlating equation for their measurements taken over 20 °C to 100 °C, and we computed values at 5 points over this range for comparisons. The deviations are shown in Fig. 13. All data are represented to within 8 %, although the EOS displays a bias and tends to give values that are not as negative as the experimental values.

## Extrapolation Behavior

**Fig. 14:**
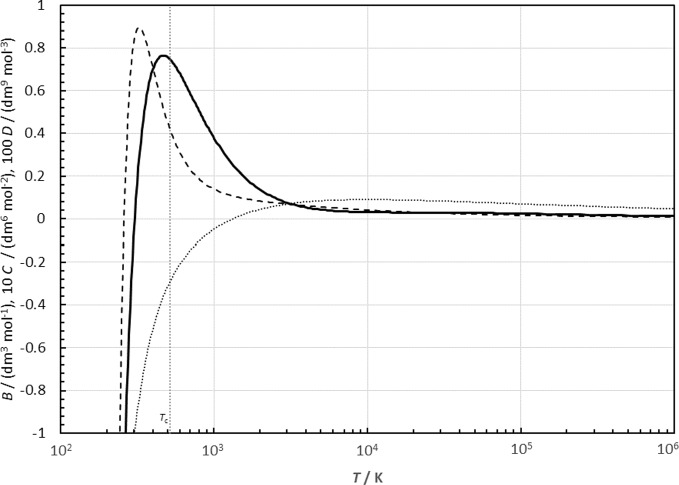
Second, third, and fourth (*B*, *C*, *D*) virial coefficients calculated from the EOS. The values of *C* and *D* are plotted as 10 *C* and 100* D*: *B* (dotted line), *C* (dashed line), *D* (solid line)

In this section, we provide a few plots of selected derived properties from the equation of state to demonstrate the limiting conditions such as when the temperature becomes very small or very large or for very high pressures, and in other extrapolated regions where there are no experimental data. Virial coefficients can be calculated from the equation of state with thermodynamic relationships as described in Lemmon and Jacobsen [17]. It is interesting to observe their behavior at limiting values of both low and high temperatures. Figure 14 shows the virial coefficients calculated from the EOS, where *B*,* C*, and *D* are the second, the third, and the fourth virial coefficients, respectively. Values of all three virial coefficients approach negative infinity as the temperature approaches zero and decrease as the temperature becomes very large, as predicted by studies on the Lennard–Jones fluid by Thol *et al.* [75]. For a Lennard–Jones fluid there should be an additional maximum at very high temperatures for *D* that was not observed in this work, but observed in their simulation studies [75].

Plots of certain curves called “characteristic curves” or “ideal curves” are useful in assessing the extrapolation behavior of an EOS in regions where data are unavailable [17, 76]. The book of Span [18] gives considerably more details. Along an ideal curve, the property of a real fluid corresponds to a hypothetical ideal fluid at the same temperature and density. When applied to the compressibility factor *Z*, these are known as [17] the Boyle curve,

17 $$ \left( {\frac{\partial Z}{{\partial v}}} \right)_{T} = 0, $$

the Joule–Thomson inversion curve,

18 $$ \left( {\frac{\partial Z}{{\partial T}}} \right)_{p} = 0, $$

the Joule inversion curve,

19 $$ \left( {\frac{\partial Z}{{\partial T}}} \right)_{v} = 0, $$

and the ideal curve,

20 $$ Z = \frac{p}{\rho RT} = 1. $$

**Fig. 15:**
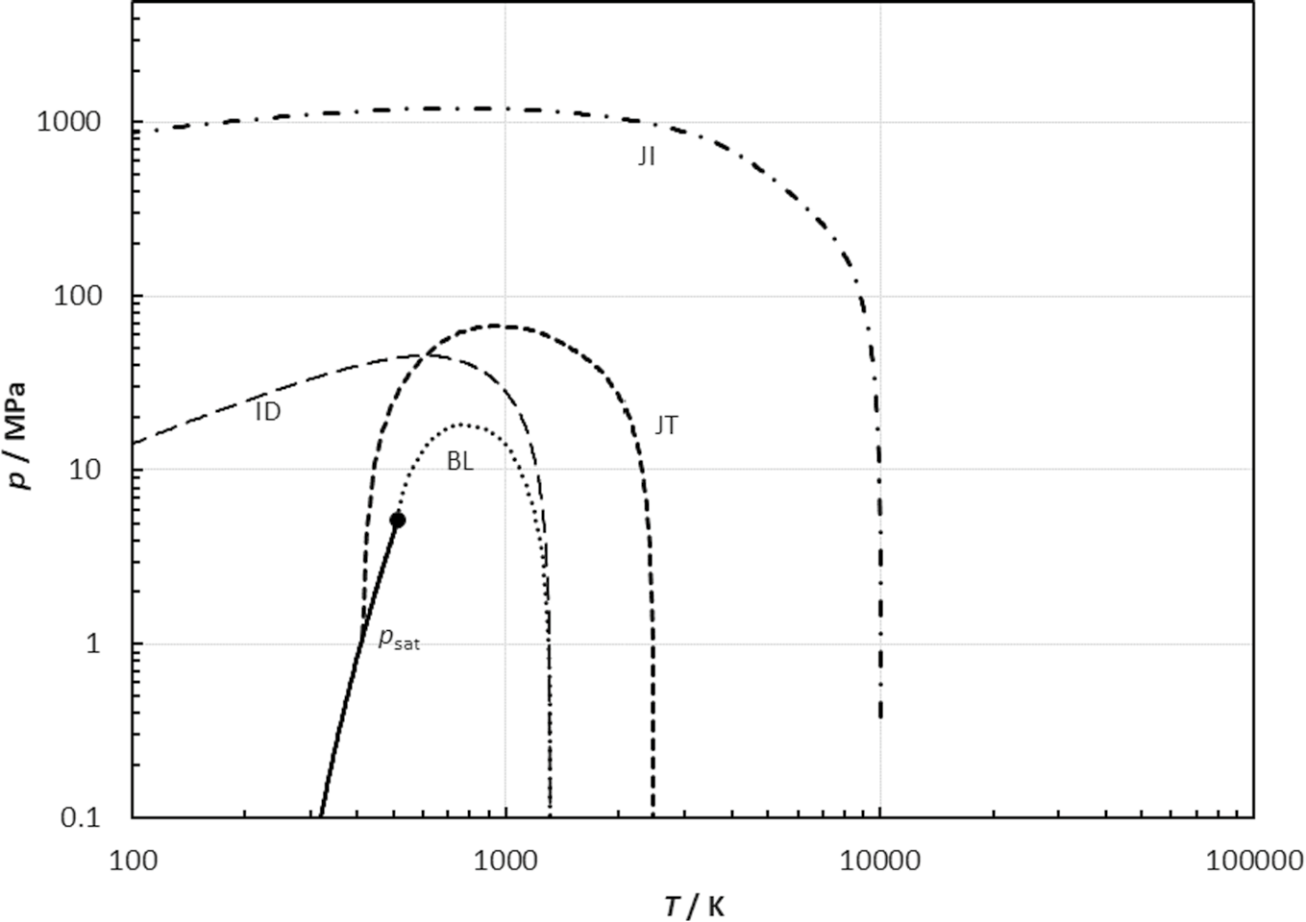
Ideal curves: the ideal curve (ID, long dashed line), the Boyle curve (BL, dotted line), the Joule–Thomson inversion curve (JT, medium dashed line), the Joule inversion curve (JI, dash-dot line), and the vapor-pressure curve (*p*_s_, solid line). The solid circle indicates the critical point

These curves, along with the vapor-pressure curve, are shown in Fig. 15. There are no large bumps or unusual changes in slope, indicating that the extrapolation behavior to extreme conditions is qualitatively correct.

**Fig. 16:**
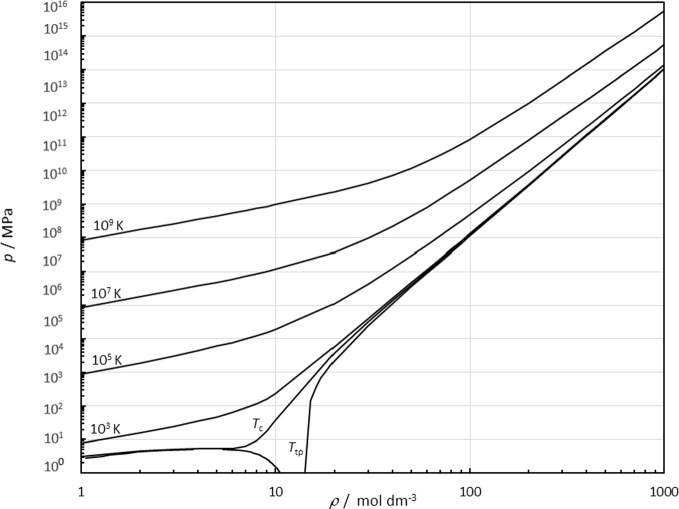
Pressure versus density along isotherms at extreme conditions

Another test of the extrapolation behavior of an EOS is to examine the behavior along isotherms at extreme conditions of temperature and density. Figure 16 shows a pressure-density plot along isotherms at extreme conditions. There is no unusual behavior evident at temperatures up to 10^9^ K and pressures of ~ 10^14^ MPa.

The phase identification parameter (PIP) is defined by Venkatarathnam and Oellrich [77] as

21 $$ {\text{PIP}} = \Pi = 2 - \rho \left[ {\frac{{\frac{{\partial^{2} p}}{\partial p\partial T}}}{{\left( {\frac{\partial p}{{\partial T}}} \right)_{\rho } }} - \frac{{\left( {\frac{{\partial^{2} p}}{{\partial \rho^{2} }}} \right)_{T} }}{{\left( {\frac{\partial p}{{\partial \rho }}} \right)_{T} }}} \right]. $$

**Fig. 17:**
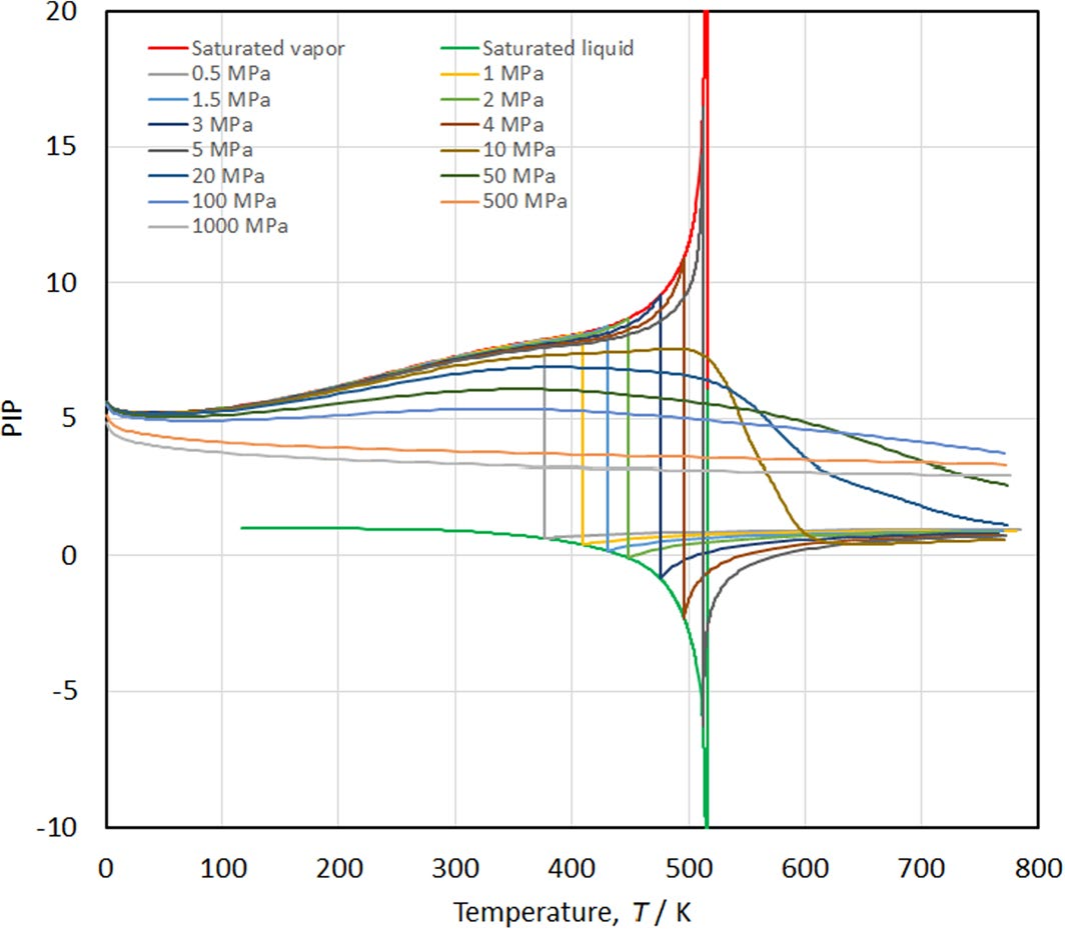
Phase identification parameter (PIP) versus temperature along isobars

It was originally developed to identify the phase of a fluid (liquid, vapor) with only partial derivatives of *p*, *v*, and *T* without reference to saturated properties. It is now often used to highlight problems in an EOS by examining plots of the PIP as a function of temperature or density and looking for unusual bumps or inflection points. Figure 17 shows the PIP versus temperature along isobars at 0.5 MPa, 1 MPa, 1.5 MPa, 2 MPa, 3 MPa, 5 MPa, 10 MPa, 20 MPa, 50 MPa, 100 MPa, 500 MPa, and 1000 MPa. The curves are all smooth, without any unreasonable behavior.

**Fig. 18:**
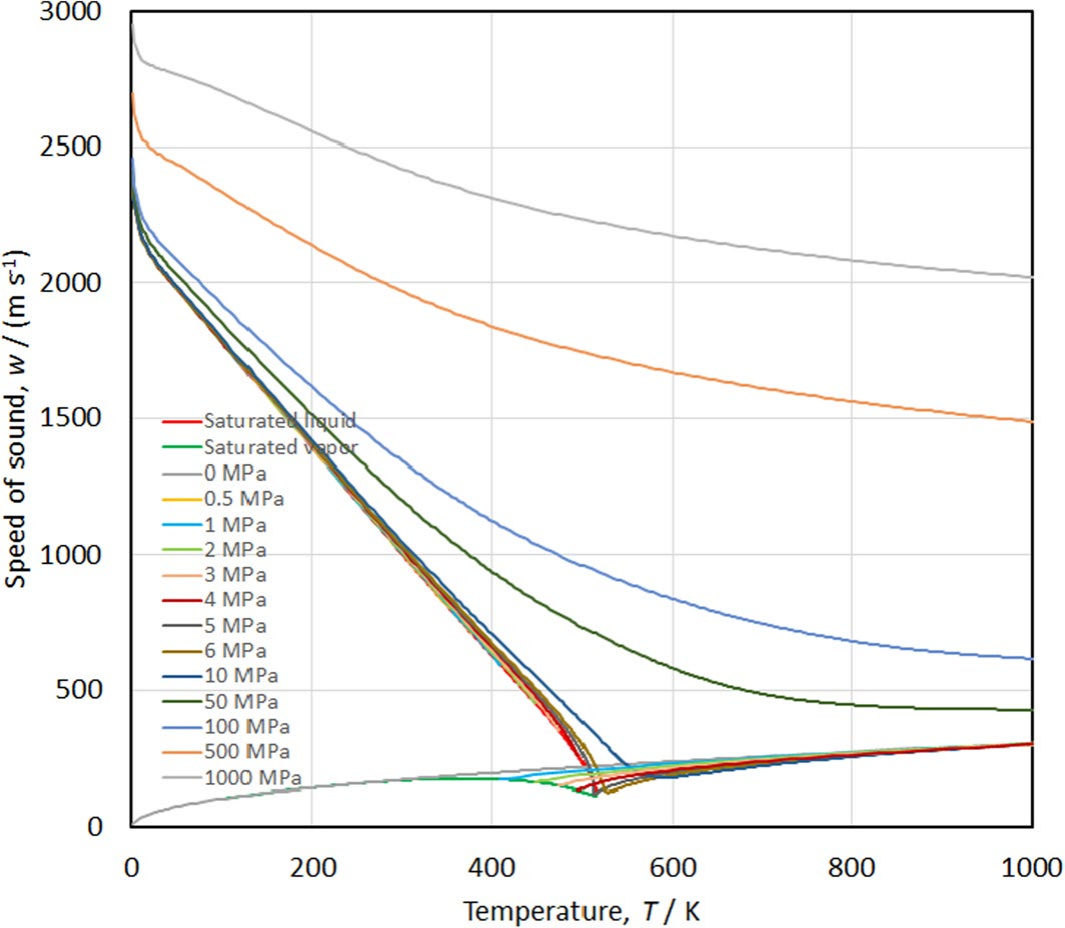
Speed of sound versus temperature diagram along isobars

**Fig. 19:**
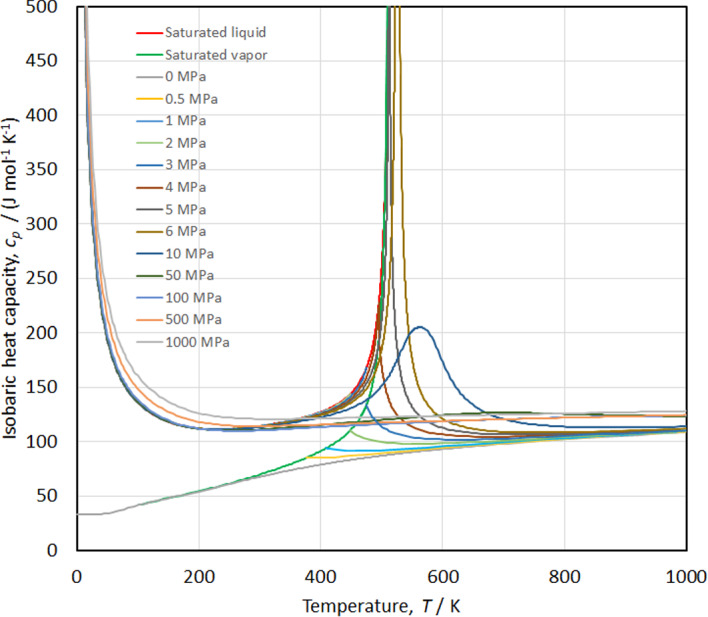
Isobaric heat capacity versus temperature diagram along isobars

**Fig. 20:**
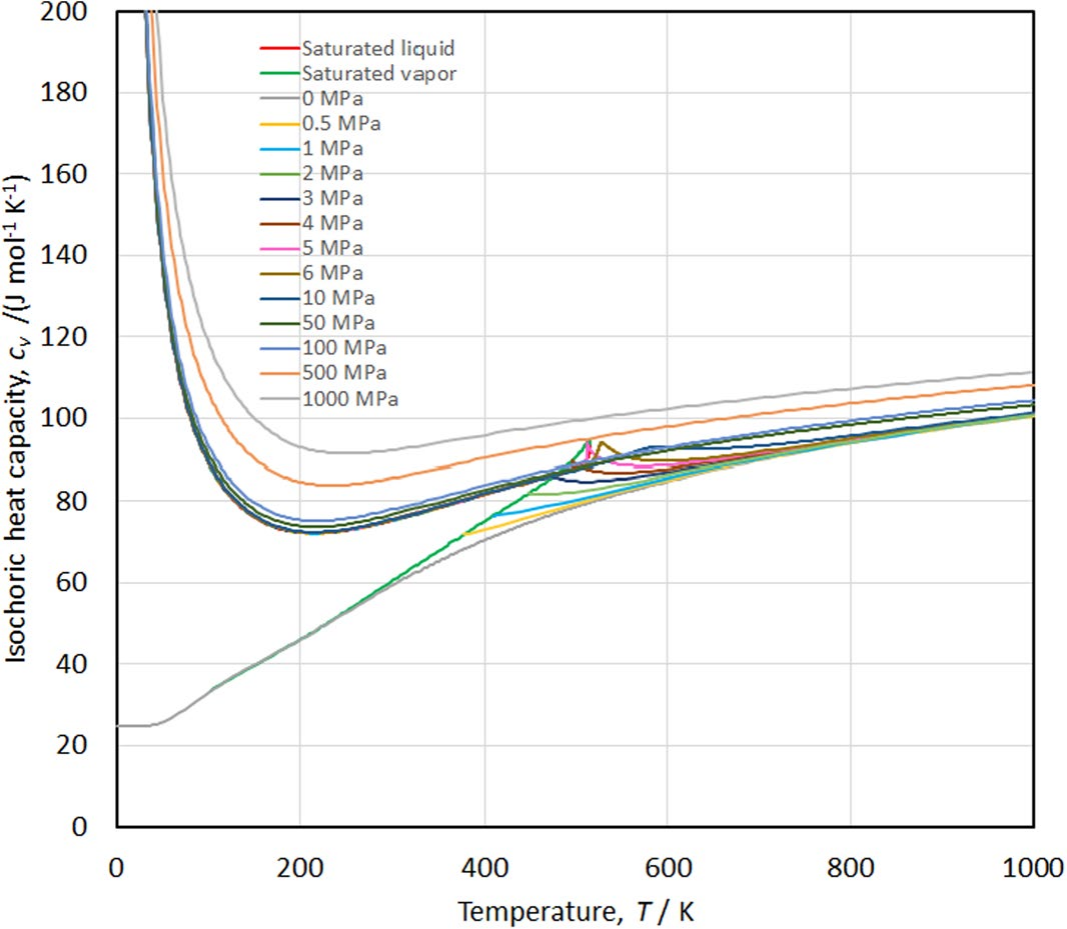
Isochoric heat capacity versus temperature diagram along isobars

**Fig. 21:**
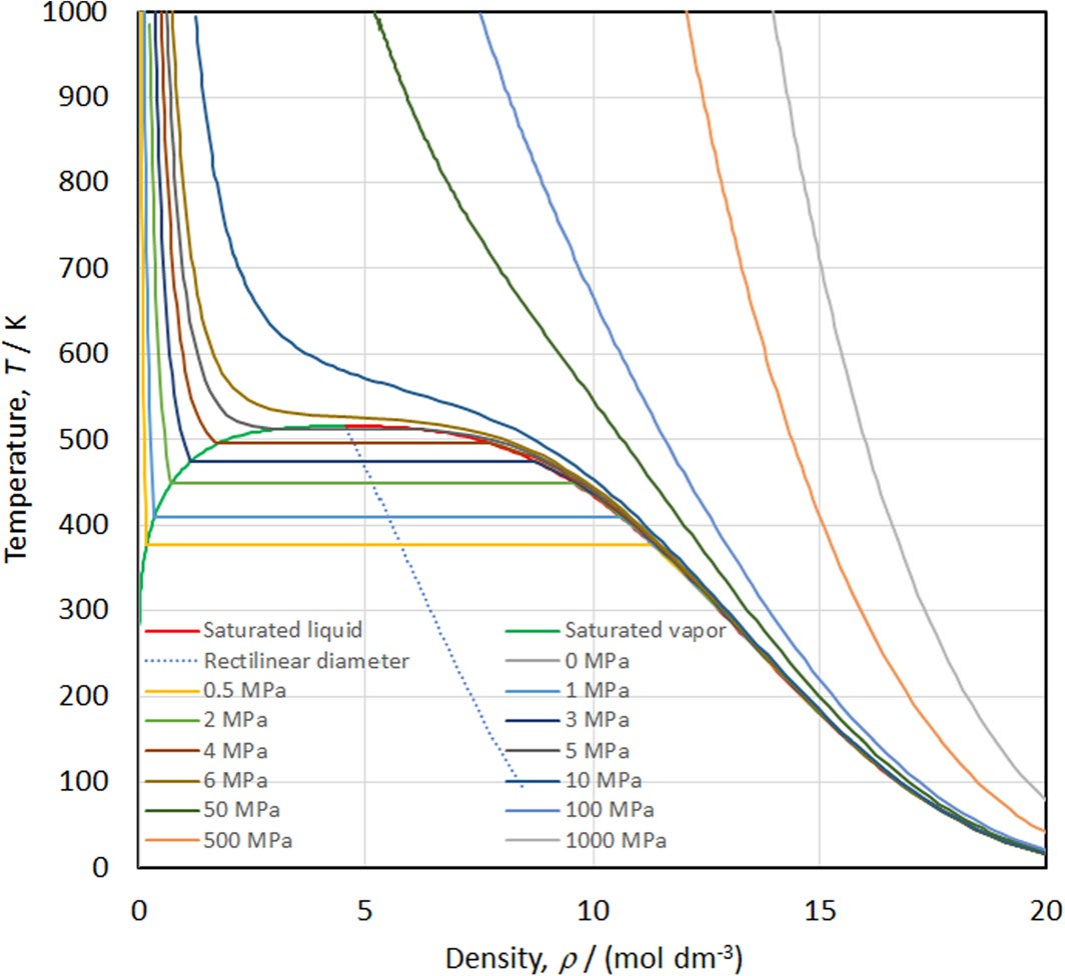
Isobaric behavior of the EOS

The speed of sound versus temperature along isobars is shown in Fig. 18, while the isobaric heat capacity and isochoric heat capacity versus temperature along isobars are shown in Figs. 19 and 20, respectively. Smooth extrapolation behavior is demonstrated in these figures with no unusual bumps or changes in slope. As noted by Akasaka and Lemmon [27] a deficiency in our EOS is that it does not exactly reproduce the theoretical critical behavior; the speed of sound should become zero and *c*_*v*_ and *c*_*p*_ should become infinite at the critical point. Figure 21 shows the temperature versus density behavior along selected isobars up to 1000 MPa. The isobars are smooth and do not cross, demonstrating physically realistic behavior.

## Discussion and Conclusions

We developed a Helmholtz energy equation of state for R-1130(E) that is explicit in temperature and density. It is valid from the triple-point temperature of 223.31 K to 525 K, and at pressures up to 30 MPa. Ancillary equations for the saturated liquid and vapor densities and for the vapor pressure have been presented that can be used for rapid approximate calculations of saturation properties or as initial values for iterative calculations with the EOS. The Helmholtz energy EOS was developed with experimental data for *pρT*, isobaric heat capacity, speed of sound, and vapor pressure. Computational chemistry was used to obtain ideal-gas heat capacities that were incorporated into the regression procedure. The estimated uncertainties (at a *k* = 2 or 95 % level of confidence) are based on comparisons with critically assessed data and are 0.25 % for vapor pressure for temperatures in the range 300 K < *T* < 454 K, rising to 1.5 % as the temperature decreases from 300 K to 265 K. For density in the liquid phase the estimated uncertainty is 0.14 % over 270 K < *T* < 410 K at pressures up to 30 MPa. For the vapor phase the estimated uncertainty in density is 3 % based on limited comparisons with the data of Tanaka *et al.* [4] that cover 343 K < *T* < 454 K. The uncertainty for liquid-phase heat capacity is 1 % at atmospheric pressure over the temperature range 268 K < *T* < 309 K, and the uncertainty for the speed of sound in the liquid phase is 0.25 % over 230 K < *T* < 420 K at pressures up to 30 MPa. The uncertainties are larger outside of these specified ranges and in the critical region. In addition, plots are provided that demonstrate that the EOS has reasonable extrapolation behavior outside of the range of experimental data.

## Supplementary Information

Below is the link to the electronic supplementary material.

Supplementary file1 (ZIP 301 KB)
The supplemental information includes a ZIP folder containing the file R1130E.FLD.txt that can be used with the NIST REFPROP [78] computer program (but must be renamed R1130E.FLD). Additional Supplemental Information includes the tabulated values of the ideal gas heat capacity, an ECS model for the viscosity and thermal conductivity for use with the EOS developed in this work, and a small Python program to check implementation of the EOS.

## Data Availability

Not applicable. No datasets were generated or analysed during the current study.
