# Supplementary material for: Equation of State for the Thermodynamic Properties of Trans-1,2-dichloroethene [R-1130(E)]
Source: Int J Thermophys. 2025 Mar 28;46(5):76. doi: 10.1007/s10765-025-03535-3 (PMC11953157; doi:10.1007/s10765-025-03535-3)
Supplement: Supplementary file 1 — Supplementary file1 (ZIP 301 KB) The supplemental information includes a ZIP folder containing the file R1130E.FLD.txt that can be used with the NIST REFPROP [78] computer program (but must be renamed R1130E.FLD). Additional Supplemental Information includes the tabulated values of the ideal gas heat capacity, an ECS model for the viscosity and thermal conductivity for use with the EOS developed in this work, and a small Python program to check implementation of the EOS. [file 10765_2025_3535_MOESM1_ESM.zip › 10765_2025_3535_MOESM1_ESM/Supplemental Information.Jan23.pdf]

Supplementary Information

Equation of State for the Thermodynamic Properties of *trans*-1,2-Dichloroethene [R-1130(E)]

Marcia L. Huber, Andrei F. Kazakov, and Eric W. Lemmon

Applied Chemicals and Materials Division, National Institute of Standards and Technology, Boulder, Colorado 80305, United States

Contents

- Ideal gas heat capacity for R-1130(E)
- Transport property models for viscosity and thermal conductivity of R-1130(E)

Table S1 Ideal gas heat capacity for R1130(E) obtained from a conventional rigid-rotor/harmonic-oscillator model with vibrational frequencies at the B3LYP-D3(BJ)/def2-TZVP level.

T [K]	c_p^o, B3LYP [J mol^{-1} K^{-1}]	*T* [K]	c_p^o, B3LYP [J mol^{-1} K^{-1}]
200	54.98	600	92.90
210	56.16	610	93.49
220	57.35	620	94.07
230	58.54	630	94.64
240	59.73	640	95.20
250	60.93	650	95.75
260	62.12	660	96.28
270	63.31	670	96.80
280	64.49	680	97.31
290	65.67	690	97.82
298.15	66.62	700	98.31
300	66.83	710	98.79
310	67.98	720	99.26
320	69.11	730	99.72
330	70.23	740	100.18
340	71.33	750	100.62
350	72.41	760	101.06
360	73.47	770	101.49
370	74.52	780	101.91
380	75.54	790	102.32
390	76.53	800	102.73
400	77.51	810	103.13
410	78.47	820	103.52
420	79.40	830	103.90
430	80.31	840	104.28
440	81.20	850	104.65
450	82.07	860	105.01
460	82.92	870	105.37
470	83.75	880	105.73
480	84.56	890	106.07
490	85.35	900	106.41
500	86.12	910	106.75
510	86.88	920	107.08
520	87.61	930	107.40
530	88.33	940	107.72
540	89.03	950	108.03
550	89.71	960	108.34
560	90.38	970	108.64
570	91.03	980	108.94
580	91.67	990	109.24
590	92.29	1000	109.52

Transport Property Models

Al-Barghouti et al. [1] recently developed extended corresponding-states (ECS) models for the viscosity and thermal conductivity of R1130(E). At that time, a Helmholtz energy equation of state was not available, so a volume-translated Peng-Robinson EOS [2] was used to obtain density. Since the density prediction in the Helmholtz energy model in this work is superior to the Peng-Robinson model, we have updated the ECS models for both the viscosity and thermal conductivity of R1130(E) for incorporation into a REFPROP v10 fluid [3] file. The extended corresponding-states model for transport properties has been described previously [4-6] and will only briefly be discussed here.

Viscosity

Implementation of the ECS models requires a Lennard-Jones collision diameter σ and a Lennard-Jones pair potential well depth ε that were estimated with the method of Chung et al. [7] that used the critical point values found in this work, resulting in σ = 0.489 nm and $\varepsilon/(k_B T)$ = 409.5 K where k_B is the Boltzmann constant. The coefficients c_k (Eq. 11 in ref.[6]) that are used to empirically correct the ECS viscosity,

$$\psi(\rho_r)=\sum_{k=0}^{1} c_k \rho_r^k ,$$

were determined by fitting the experimental data of Awbery and Griffiths [8] and Ketelaar et al. [9] to a linear function in reduced density ($\rho_r=\rho/\rho_c$), resulting in parameter values of c_0 = 1.03058 and c_1 = –0.0285132. The reference fluid used was R-134a [10-12]. The results are shown in Figure S1. All viscosities are in the liquid phase at atmospheric pressure; no vapor data were found. The data are represented to within 3.7%.

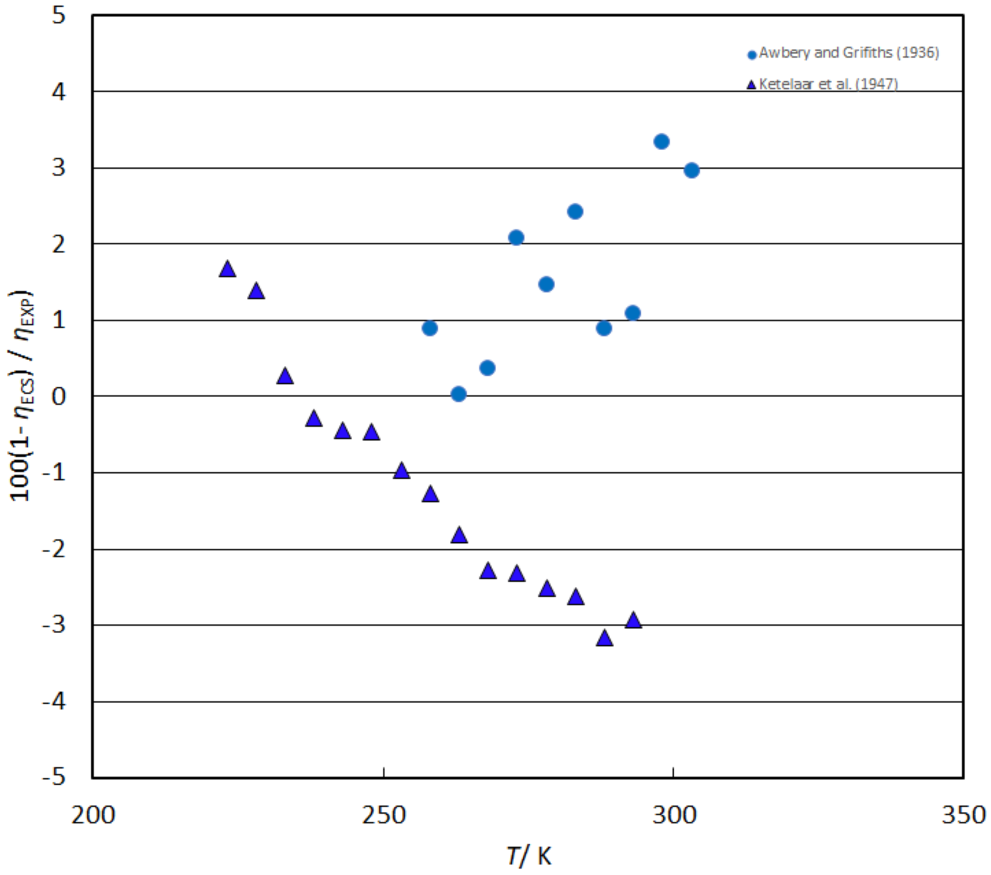

Figure S1. Comparisons of viscosity predicted by the ECS model with experimental data.

A.2.2 Thermal Conductivity

An ECS model as described in Huber [6] was used to fit the thermal conductivity data of Al-Barghouti et al. [1]. The resulting coefficients for Eq. (21) in Huber [6],

$$\chi(\rho_{\mathrm{r}}) = \sum_{k=0}^{1} b_k \rho_{\mathrm{r}}^k ,$$

were found to be $b_0 =$ 0.98257 and $b_1 = -0.0045654$ with the use of a linear fit in reduced density. For the dilute gas thermal conductivity, coefficient f_{int} (Eq. 16 in ref. [6]) was set to a constant value 0.00125 $\mathrm{W \cdot m^{-1} \cdot K^{-1}}$. The critical enhancement of thermal conductivity was estimated with the method of Perkins et al. [13]. Figure S2 shows the results of the thermal conductivity fit with the ECS model. The expanded estimated uncertainty (k = 2) is the same as the experimental data, 1.4% for the range of the experimental data, 240 K to 340 K at pressures up to 25 MPa.

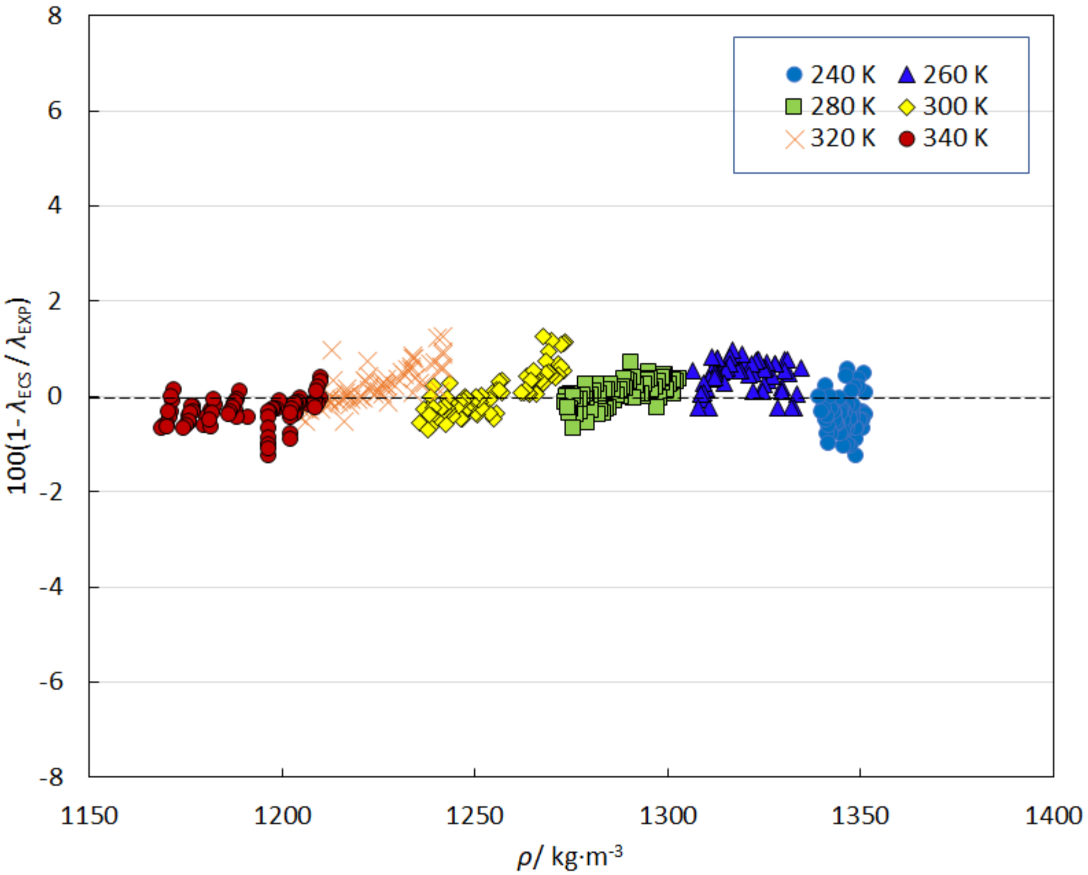

Figure S2. Comparisons of thermal conductivity predicted by the ECS model with experimental data.

REFERENCES

1. K.S. Al-Barghouti, A.J. Rowane, I.H. Bell, M.L. Huber, R.A. Perkins, Int. J. Thermophys. 45 (3), 36 (2024). https://doi.org/10.1007/s10765-024-03334-2
2. A. Péneloux, E. Rauzy, R. Fréze, Fluid Phase Equilib. 8 (1), 7 (1982). https://doi.org/10.1016/0378-3812(82)80002-2
3. E.W. Lemmon, I.H. Bell, M.L. Huber, M.O. McLinden, NIST Standard Reference Database 23: Reference Fluid Thermodynamic and Transport Properties-REFPROP, Version 10.0, National Institute of Standards and Technology, Standard Reference Data Program, Gaithersburg (2018). https://doi.org/10.18434/T4/1502528
4. M.O. McLinden, S.A. Klein, R.A. Perkins, Int. J. Refrig. 23 (1), 43 (2000). https://doi.org/10.1016/S0140-7007(99)00024-9
5. S.A. Klein, M.O. McLinden, A. Laesecke, Int. J. Refrig. 20 (3), 208 (1997). https://doi.org/10.1016/S0140-7007(96)00073-4
6. M.L. Huber, Models for the Viscosity, Thermal Conductivity, and Surface Tension of Selected Pure Fluids as Implemented in REFPROP v10.0, NISTIR 8209, 2018. https://doi.org/10.6028/NIST.IR.8209

7. T.H. Chung, M. Ajlan, L.L. Lee, K.E. Starling, Ind. Eng. Chem. Res. 27 (4), 671 (1988). https://doi.org/10.1021/ie00076a024
8. J.H. Awbery, E. Griffiths, Proc. Phys. Soc. 48, 372 (1936). https://doi.org/10.1088/0959-5309/48/3/303
9. J.A.A. Ketelaar, L. De Vries, P.F. Van Velden, J.S. Kooy, Recl. Trav. Chim. Pays-Bas 66, 733 (1947).
10. R. Tillner-Roth, H.D. Baehr, J. Phys. Chem. Ref. Data 23 (5), 657 (1994). https://doi.org/10.1063/1.555958
11. R.A. Perkins, A. Laesecke, J. Howley, M.L.V. Ramires, A.N. Gurova, L. Cusco, NISTIR 6605 (2000). https://doi.org/10.6028/NIST.IR.6605
12. M.L. Huber, A. Laesecke, R.A. Perkins, Ind. Eng. Chem. Res. 42 (13), 3163 (2003). https://doi.org/10.1021/ie0300880
13. R.A. Perkins, J.V. Sengers, I.M. Abdulagatov, M.L. Huber, Int. J. Thermophys. 34 (2), 191 (2013). https://doi.org/10.1007/s10765-013-1409-z
